# Design and Synthesis of a Novel Ganglioside Ligand for Influenza A Viruses ^†^

**DOI:** 10.3390/molecules17089590

**Published:** 2012-08-10

**Authors:** Tomohiro Nohara, Akihiro Imamura, Maho Yamaguchi, Kazuya I. P. J. Hidari, Takashi Suzuki, Tatsuya Komori, Hiromune Ando, Hideharu Ishida, Makoto Kiso

**Affiliations:** 1 Department of Applied Bioorganic Chemistry, Gifu University, 1-1 Yanagido, Gifu-shi, Gifu 501-1193, Japan; Email: noharah18@yahoo.co.jp (T.N.); komorih17@yahoo.co.jp (T.K.); hando@gifu-u.ac.jp (H.A.); ishida@gifu-u.ac.jp (H.I.); 2 Institute for Integrated Cell-Material Sciences, Kyoto University, 69 Konoe-cho, Yoshida, Sakyo-ku, Kyoto 606-8501, Japan; 3 Department of Biochemistry, School of Pharmaceutical Sciences, University of Shizuoka, 52-1 Yada, Suruga-ku, Shizuoka-shi, Shizuoka 422-8526, Japan; Email: d11107@u-shizuoka-ken.ac.jp (M.Y.); hidari@u-shizuoka-ken.ac.jp (K.I.P.J.H.); suzukit@u-shizuoka-ken.ac.jp (T.S.)

**Keywords:** ganglioside, sialic acid, total synthesis, glycosylation, influenza virus

## Abstract

A novel ganglioside bearing Neuα2-3Gal and Neuα2-6Gal structures as distal sequences was designed as a ligand for influenza A viruses. The efficient synthesis of the designed ganglioside was accomplished by employing the cassette coupling approach as a key reaction, which was executed between the non-reducing end of the oligosaccharide and the cyclic glucosylceramide moiety. Examination of its binding activity to influenza A viruses revealed that the new ligand is recognized by Neuα2-3 and 2-6 type viruses.

## 1. Introduction

**Figure 1:**
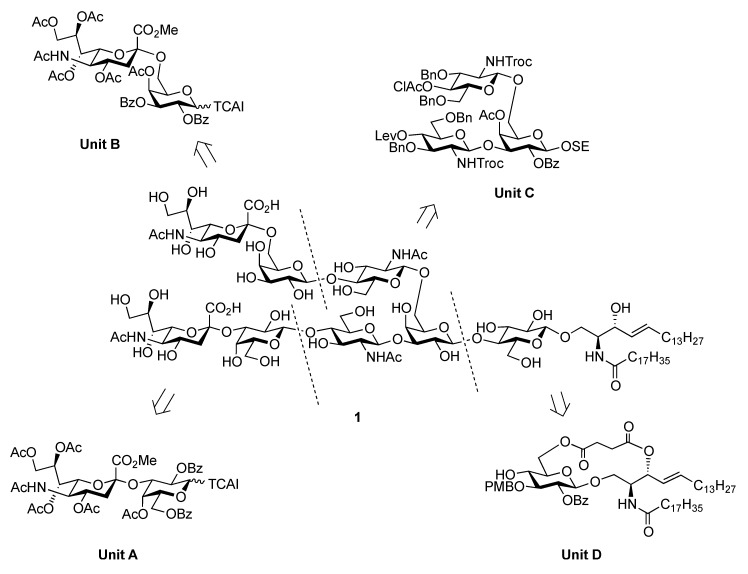
Structures of the target ganglioside **1** and the designed building blocks (**Units A**–**D**).

Influenza viruses cause a substantial number of deaths during annual epidemics and occasional pandemics [1,2]. Based on the antigenicity of their internal proteins the viruses are divided into three types, A, B, and C, of which either type A or B viruses cause seasonal influenza in humans. When the viruses bind to the host cell, hemagglutinin (HA) on their cell surface plays a significant role in the infection process. The HA protein recognizes sialoglycoconjugates expressed on the plasma membrane of the host cell, for example, sialoglycoproteins and gangliosides (sialoglycosphingolipids), as cellular ligands. Furthermore, HA can also recognize specific linkages between sialic acid (Neu5Ac/Gc) and lactosamine (LacNAc: Galβ1-4GlcNAc) residues, which are found at the terminal end of glycoconjugates [3,4]. The structure and distribution of sialoglycans are crucial for viruses to determine their host animals, and two major linkage types, that is, Neu5Acα2-3LacNAc and Neu5Acα2-6LacNAc, are essential for viral transmission. Human and swine viruses predominantly recognize the Neu5Acα2-6LacNAc sequence, while avian and equine viruses bind preferentially to the Neu5Acα2-3Gal (including Neu5Acα2-3LacNAc) moiety. Swine are considered as intermediate hosts between humans and birds since they possess an abundance of both Neu5Acα2-3LacNAc and Neu5Acα2-6LacNAc structures as receptor carbohydrate determinants. The simultaneous infection of an intermediate host, such as swine, with avian and human viruses could lead to genetic recombination between the viruses, resulting in the generation of a new pathogenic virus that could potentially cause severe pandemics. However, the exact natural ligand for influenza A viruses in an intermediate host, such as pigs, remains unclear. Therefore, in this study, we focused on identifying a new carbohydrate ligand that was not only highly recognized by influenza A viruses but also functions as a natural receptor for viral HA. For this purpose, a ganglioside bearing both the Neu5Acα2-3 and Neu5Acα2-6LacNAc sequences was designed (Figure 1). It was hypothesized that the designed ganglioside **1** could be recognized by human- and avian-derived viruses because it contains two types of sialoglycan in a single molecule. We report the chemical synthesis of ganglioside **1** and its binding activity to influenza A viruses.

## 2. Results and Discussion

### 2.1. Chemical Synthesis

It was envisaged that the efficient synthesis of **1** could be achieved using the cassette approach between the non-reducing end of the oligosaccharide and the glucosylceramide, which was recently developed by our group [5,6,7,8,9,10]. Furthermore, it was thought that the construction of the non-reducing end of the heptasaccharide moiety, which includes two types of sialoside, Neu5Acα2-3/2-6Gal, should be executed through a convergent synthetic approach. For the convergent synthesis of a relatively large oligosaccharide such as **1**, the design of the building blocks often affects the efficiency of the total synthesis as well as the overall yield. Our preliminary experiment on the synthesis of a ganglioside similar to **1** gave a significant finding that monosaccharyl (GlcN) units are more useful as glycosyl donors than oligosaccharyl (Neu5Acα2-3/2-6Galβ1-4GlcN) donors for the formation of branched structure at the 3- and 6-positions of the inner galactose residue (data not shown). Therefore, target **1** was divided into four major components, from which each building block (**Units A**–**D**) was designed (Figure 1). 

**Scheme 1:**
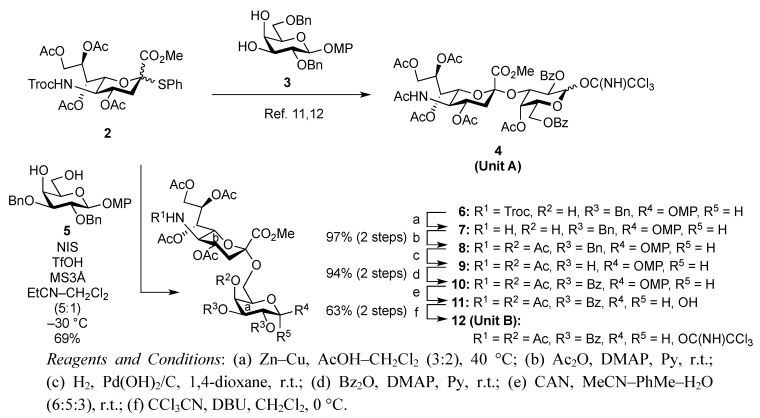
Efficient synthesis of **Units A** and **B** from the *N*-Troc-protected sialyl donor **2**.

The synthetic method for the terminal Neu5Acα2-3Gal unit **A** has been already established by our group. The coupling of the 5-*N*-Troc-protected sialyl donor **2** and galactosyl acceptor **3**, carrying a *p*-methoxyphenyl (MP) group at the anomeric position, generated the Neu5Trocα2-3Gal disaccharide in good yield. The isolation of α-sialoside from the reaction mixture was easily accomplished by recrystallization [11]. The obtained disaccharide was readily converted into the corresponding trichloroacetimidate donor as **Unit A** [12]. Similarly, the other terminal Neu5Acα2-6Gal unit (**B**) was prepared efficiently according to the synthetic procedure for **Unit A** (Scheme 1). The sialylation of the diol galactosyl acceptor **5** was performed in the presence of NIS and TfOH [13,14] in a mixed solvent system, propionitrile–dichloromethane (5:1), at −30 °C [15,16]. This mixed solvent system was used because of the poor solubility of the acceptor **5** in acetonitrile. In addition, temperatures lower than −30 °C led to a significant decrease in the yield, possibly because of the observed precipitation of **5** during the reaction. As a result of optimization, the desired α-glycoside **6** was obtained in 69% yield along with a 14% yield of the β-isomer. Purification of the α-glycoside **6** by silica gel column chromatography was troublesome compared with that of the regioisomer, Neu5Trocα2-3GalMP, which has benzyl groups on the O-2 and O-6 positions of its galactose residue, which can be isolated easily by recrystallization from an EtOAc/*n*-hexane system [12]. The selective deprotection of the Troc group with Zn–Cu [17] (giving **7**) and the subsequent concomitant acetylation of the liberated amine and the hydroxyl group at the C-4 position of the galactose residue afforded compound **8** in excellent yield. Hydrogenolysis of the benzyl groups over Pearlman’s catalyst in 1,4-dioxane followed by benzoylation generated compound **10** in 94% yield over two steps. The exposure of **10** to CAN and H_2_O [18] (giving **11**) and the subsequent introduction of a trichloroacetimidate group [19] afforded the Neu5Acα2-6Gal donor **12** (**Unit B**) in 63% yield over two steps.

**Scheme 2:**
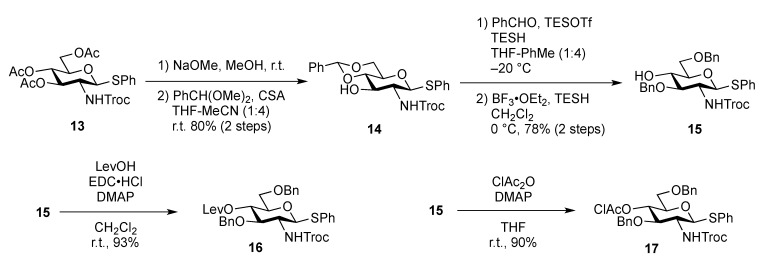
Preparation of glucosaminyl donors **16** and **17** towards **Unit C**.

**Scheme 3:**
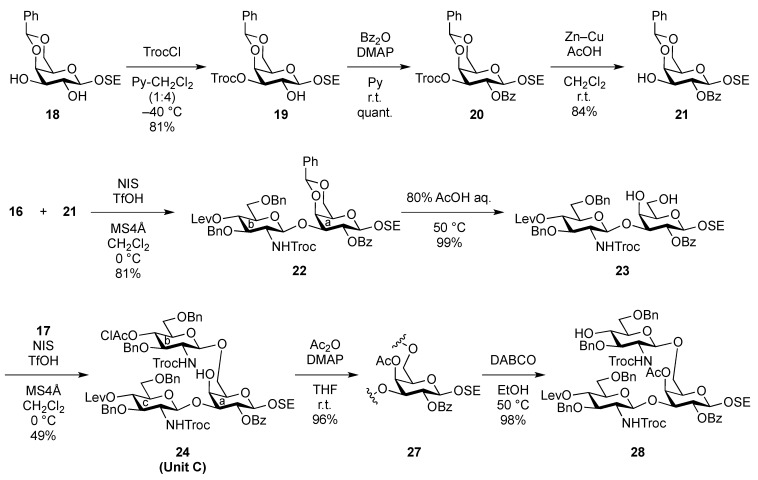
Assembly of the inner core fragment, **Unit C**.

The inner core trisaccharide structure, **Unit C**, was prepared starting from the 2-*N*-Troc protected glucosamine derivative **13** (Scheme 2 and Scheme 3). First, the glucosaminyl donors **16** and **17** were prepared as shown in Scheme 2. Removal of the acetyl groups from **13** and the subsequent formation of cyclic benzylidene acetal between O-4 and O-6 afforded **14** in good yield. The following benzylation step in the presence of a Troc group was conducted under reductive conditions. Optimization of this reductive benzylation with benzaldehyde, TESOTf, and triethylsilane [20] revealed that the use of toluene as a solvent could increase the yield. The successive reductive opening of the benzylidene group by treatment with BF_3_ etherate and triethylsilane [21] gave **15** in 78% yield over two steps from **14**. The obtained alcohol **15** was transformed into two types of glucosaminyl donors, namely, **16** and **17**, via the introduction of a levulinoyl (Lev) and monochloroacetyl (ClAc) group to the hydroxyl group at C-4, respectively.

**Figure 2:**
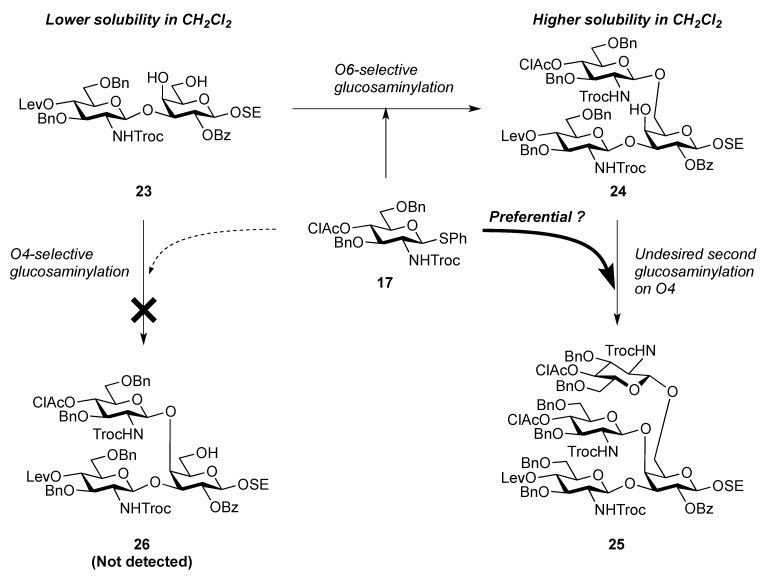
Explanation for the poor regioselectivity observed during glucosaminylation.

The glucosaminyl donors **16** and **17** were then incorporated into the galactosyl acceptor **21**, which was prepared readily from the known galactose derivative **18** [22] via three steps. Selective protection of the hydroxyl group at C-3 by the Troc group was carried out under basic conditions with TrocCl at a low temperature (−40 °C) to afford **19** in 81% yield. Benzoylation under standard conditions (giving **20**) and the selective removal of the Troc group with Zn–Cu furnished the galactosyl acceptor **21** in good yield. The obtained **21** was then subjected to glycosylation with donor **16** in the presence of NIS and TfOH in CH_2_Cl_2_ at 0 °C, affording the disaccharide **22** in 81% yield. The stereochemical assignment was confirmed by ^1^H-NMR, where the *J*_1,2_ value of 7.6 Hz for H-1 indicated the β-configuration of the glucosamine residue. Next, hydrolysis of the benzylidene group with 80% AcOH aq at 50 °C afforded the diol **23** at an almost quantitative yield. A second round of glucosaminidation was conducted between **17** and **23** under the same conditions as those of the initial glucosaminidation between **16** and **21**. As a result, the desired trisaccharide **24** was obtained as **Unit C** in a moderate yield of 49%. In this reaction, a non-negligible amount of the tetrasaccharide **25**, in which both hydroxyl groups were glucosaminylated, was observed as a byproduct (Figure 2). An attempt at using a lower temperature to increase the selectivity failed due to the poor solubility of acceptor **23** in CH_2_Cl_2_. Furthermore, changing the other factors for glycosylation, for example, the leaving group (using trichloroacetimidate) and how the donor was added, did not improve the yield of **24**. It is of importance that the generation of the tetrasaccharide **25** during the reaction was faster than the complete consumption of the acceptor **23**. In addition, the trisaccharide **26**, which was glucosaminylated at C-4 of the galactose residue, was not detected among the by-products. These findings suggested that the newly formed trisaccharyl alcohol **24** was preferred to the disaccharyl acceptor **23** as a glycosyl acceptor. This phenomenon might be explained by the poor solubility of the disaccharyl alcohol **23** in CH_2_Cl_2_ compared with the trisaccharyl alcohol **24** (Figure 2). Next, the acetylation of **24** with acetic anhydride and DMAP in THF [23] was carried out to protect the free hydroxyl group, affording **27** in 96% yield (Scheme 3). The monochloroacetyl group on **27** was then unblocked using DABCO in ethanol [24] with an excellent yield, providing the inner core trisaccharide acceptor **28**, which was ready for the next glycosylation step.

**Scheme 4:**
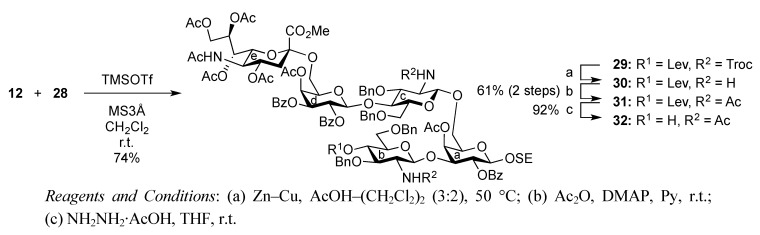
Coupling of **Units A** and **C** followed by transformation of the corresponding acceptor.

As depicted in Scheme 4, the coupling of the trisaccharide acceptor **28** with the Neu5Acα2-6Gal donor **12** promoted by TMSOTf was conducted in CH_2_Cl_2_ at room temperature, affording the pentasaccharide **29** in 74% yield. During this glycosylation step, the generation of several by-products containing trichloroacetamide glycoside, which is occasionally formed as a by-product during glycosylation using trichloroacetimidate donors, made the purification process an arduous task. Column chromatography on silica gel followed by gel filtration was found to be useful for purification. Next, the conversion of the Troc carbamate at C-2 of both glucosamine residues into acetamide was achieved by treatment of alloyed zinc with copper in AcOH and followed by acetylation, giving the acetamide compound **31** in 61% yield over two steps. Finally, cleavage of the levulinoyl group by using hydrazine monoacetate in THF [25] released the 4-OH to provide the pentasaccharide acceptor **32** in 92% yield.

**Scheme 5:**
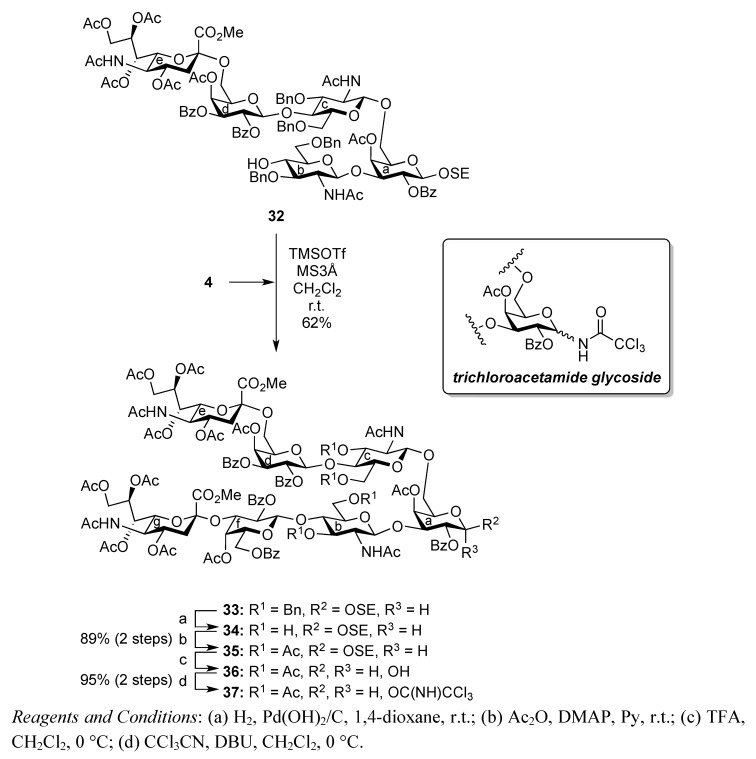
Synthesis of the non-reducing end heptasaccharide part.

Scheme 5 shows the assembly of the non-reducing end heptasaccharide moiety. The Neu5Acα2-3Gal donor **4** was coupled with **32** in the presence of TMSOTf in CH_2_Cl_2_ at room temperature to provide the heptasaccharide **33** in 62% yield. During this glycosylation step, the generation of the trichloroacetamide glycoside and the dimer of donor **4**, which was formed by the nucleophilic attack of the hydrolyzed donor on the oxocarbenium species derived from the donor, as by-products, made the purification of the desired product **33** laborious. The structure of isolated **33** was elucidated based on its MS, ^1^H, and ^13^C-NMR spectra. For instance, the β-configuration of the newly formed glycosidic linkage was evident from the coupling constant of the anomeric proton at δ 5.01 (*J*_1,2_ = 7.5 Hz). Next, cleavage of the benzyl groups by hydrogenolysis (giving **34**) followed by acetylation with conventional conditions afforded **35** in 89% yield over two steps. Selective exposure of the anomeric hydroxyl group was easily achieved by treatment with trifluoroacetic acid in CH_2_Cl_2_ to yield **36**. This was then converted to the corresponding trichloroacetimidate donor **37** in 95% yield over two steps from **35**, which was then ready for cassette coupling with the glucosylceramide block **38** (**Unit D**).

**Scheme 6:**
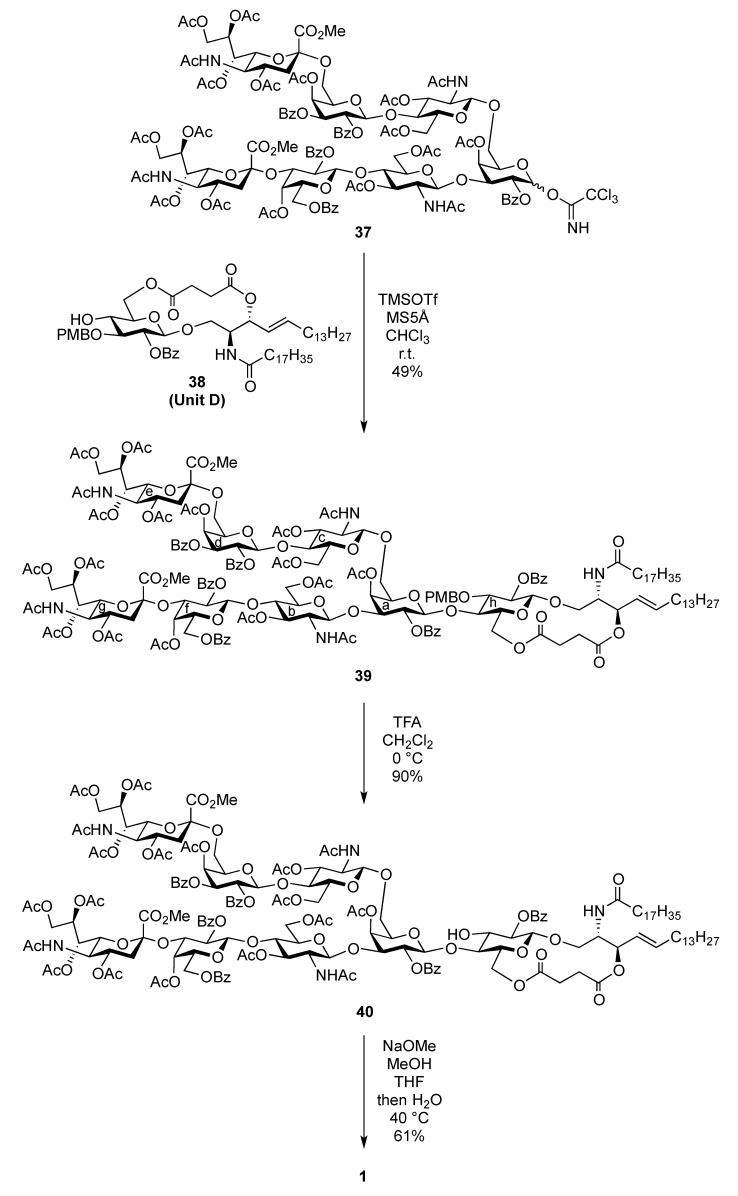
Final coupling by the cassette approach followed by global deprotection.

We previously addressed the development of the cassette coupling approach between a non-reducing end oligosaccharide and a glucosylceramide (GlcCer) moiety for the synthesis of various glycolipids, particularly gangliosides [5,6,7,8,9,10]. This approach resulted in a solution to the inevitable low yield of sugar and ceramide fragments. Hitherto, we developed two types of GlcCer units: one is a cyclic type GlcCer tethered by succinic ester between the sugar and lipid portions [5,7,8], while the other is an acyclic type GlcCer [6,9,10]. In this study, we chose the cyclic type due to its ease of preparation. The reported cyclic GlcCer acceptor **38** [7] was subjected to glycosylation with the oligosaccharide donor **37** in the presence of TMSOTf in CHCl_3_ at room temperature, affording the fully protected ganglioside **39** in a moderate yield of 49%. In this reaction, chloroform was employed as solvent instead of the conventional dichloromethane because of the somewhat poor solubility of **38** in CH_2_Cl_2_. Following the same protocol as that used for the other glycosylation reactions, the structure of **39** was elucidated. Next, cleavage of the *p*-methoxybenzyl (PMB) group by TFA in CH_2_Cl_2_ at 0 °C provided **40** in 90% yield. Finally, global deprotection under Zemplén conditions followed by saponification generated the target ganglioside **1** in good yield (Scheme 6).

### 2.2. Binding Assay

**Figure 3:**
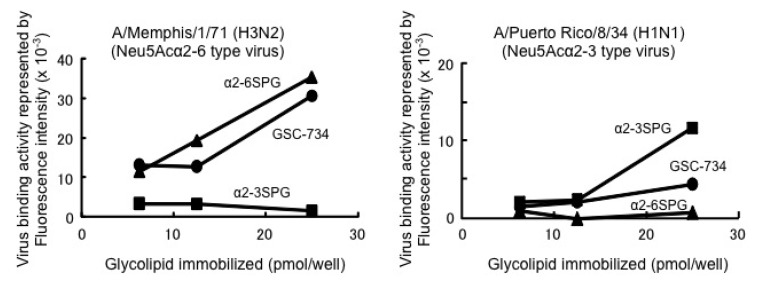
Binding activity of the synthetic ligand (**GSC-734**) for influenza A viruses.

The synthesized ganglioside **1** (termed **GSC-734**) was then assessed for its binding activity to influenza viruses (Figure 3). The binding assay showed that GSC-734 was recognized by Neu5Acα2-3 and 2-6 type viruses. Moreover, the binding activity of the Neu5Acα2-6 type virus (H3N2) was almost identical to that of the previously reported α2-6 sialylparagloboside [26]. This observation suggests that the branched structure of the sugar part does not potently influence its binding activity to the viruses. 

## 3. Experimental

### 3.1. General Methods for Chemical Synthesis

All reactions were carried out under a positive pressure of argon, unless otherwise noted. All chemicals were purchased from commercial suppliers and used without further purification, unless otherwise noted. Molecular sieves were purchased from Wako Chemicals Inc. (Osaka, Japan) and dried at 300 °C for 2 h in a muffle furnace prior to use. Solvents as reaction media were dried over molecular sieves and used without purification. TLC analysis was performed on Merck TLC (silica gel 60F254 on glass plate, Darmstadt, Germany). Compound detection was either by exposure to UV light (2536 Å) or by soak in a solution of 10% H_2_SO_4_ in ethanol followed by heating. Silica gel (80 mesh and 300 mesh) manufactured by Fuji Silysia Co. (Kasugai, Japan) was used for flash column chromatography. Quantity of silica gel was usually estimated as 100 to 150-fold weight of sample to be charged. Solvent systems in chromatography were specified in *v*/*v*. Evaporation and concentration were carried out *in vacuo*. ^1^H-NMR and ^13^C-NMR spectra were recorded with JEOL ECA 400/500/600 spectrometers. Chemical shifts in ^1^H-NMR spectra are expressed in ppm (δ) relative to the signal of Me_4_Si, adjusted to δ 0.00 ppm. Data are presented as follow: Chemical shift, multiplicity (s = singlet, d = doublet, t = triplet, dd = double of doublet, dt = double of triplet, m = multiplet and/or multiple resonances), integration, coupling constant in Hertz (Hz), position of the corresponding proton. COSY methods were used to confirm the NMR peak assignments. MALDI-TOF mass spectra were run in a Bruker Autoflex (Billerica, MA, USA) and CHCA was used as the matrix. High-resolution mass (ESI-TOF MS) spectra were run in a Bruker micrOTOF. Optical rotations were measured with a ‘Horiba SEPA-300’ high-sensitive polarimeter (Kyoto, Japan). 



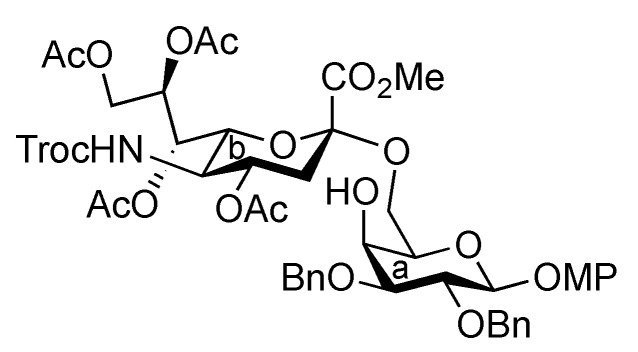



*4-Methoxyphenyl (methyl 4,7,8,9-tetra-O-acetyl-3,5-dideoxy-5-(2,2,2-trichloroethoxycarbamoyl)-D-glycero-α-D-galacto-2-nonulopyranosylonate)-(2→6)-2,3-di-O-benzyl-β-D-galactopyranoside* (**6**). To a mixture of **2** (691 mg, 0.964 mmol) and **5** (300 mg, 0.643 mmol) in EtCN/CH_2_Cl_2_ (5:1, 9.6 mL) was added 3 Å molecular sieves (991 mg) at r.t. After stirring for 1 h and then cooling to −30 °C, NIS (324 mg, 1.44 mmol) and TfOH (12.7 μL, 0.144 mmol) were added to the mixture. After stirring for 45 min at the same temperature as the reaction was monitored by TLC (1:3 EtOAc–toluene, twice development), the reaction was quenched by the addition of triethylamine. The precipitate was filtered through Celite. The filtrate was evaporated to remove EtCN and then diluted with CHCl_3_, washed with satd aq Na_2_S_2_O_3_ and brine. The organic layer was subsequently dried over Na_2_SO_4_, concentrated and the residue was purified by silica gel column chromatography (1:5 EtOAc–toluene) to give **6** (473 mg, 69%) along with its β-isomer (96 mg, 14%). [α]_D_ −13.9° (c 0.4, CHCl_3_); ^1^H-NMR (500 MHz, CDCl_3_) δ 7.38–6.80 (m, 14 H, Ar), 5.39 (m, 1 H, H-8b), 5.36 (dd, 1 H, *J*_6,7_ = 1.7 Hz, H-7b), 5.00 (d, 1 H, *J*_gem_ = 10.2 Hz, OCH_2_), 4.99 (m, 1 H, H-4b), 4.89 (d, 1 H, *J*_gem_ = 12.2 Hz, OCH_2_), 4.86 (d, 1 H, *J*_5,NH_ = 9.7 Hz, NH), 4.84 (d, 1 H, *J*_1,2_ = 8.0 Hz, H-1a), 4.83 (d, 1 H, *J*_gem_ = 10.2 Hz, OCH_2_), 4.76 (2 d, 2 H, *J*_gem_ = 12.0 Hz, OCH_2_), 4.47 (d, 1 H, *J*_gem_ = 12.2 Hz, OCH_2_), 4.32 (dd, 1 H, H-9b), 4.19 (dd, 1 H, *J*_6,7_ = 1.7 Hz, H-6b), 4.10 (dd, 1 H, H-9'b), 4.07 (near d, 1 H, H-4a), 3.94–3.89 (m, 2 H, H-2a, H-5a), 3.81–3.75 (m, 7 H, H-6a, 2 OMe), 3.65–3.61 (m, 2 H, H-6'a, H-5b), 3.57 (dd, 1 H, H-3a), 2.67–2.63 (m, 2 H, H-3b*eq*, OH), 2.12–1.99 (m, 12 H, 4 Ac), 1.91 (t, 1 H, H-3b*ax*); ^13^C-NMR (100 MHz, CDCl_3_) δ 193.2, 191.4, 170.7, 170.3, 170.1, 169.9, 167.9, 155.2, 154.0, 151.7, 138.4, 137.9, 129.0, 128.4, 128.3, 128.2, 128.1, 127.8, 127.7, 127.6, 125.3, 118.6, 114.4, 102.9, 98.7, 95.3, 80.6, 78.7, 77.2, 75.3, 74.5, 72.6, 72.3, 72.2, 68.6, 68.4, 67.5, 66.0, 63.1, 62.3, 55.6, 53.0, 51.6, 37.5, 29.7, 29.5, 21.4, 21.0, 20.8, 20.7. *m/z* (MALDI): found [M+Na]^+^ 1094.32, C_48_H_56_Cl_3_NO_20_calcd for [M+Na]^+^ 1094.32.



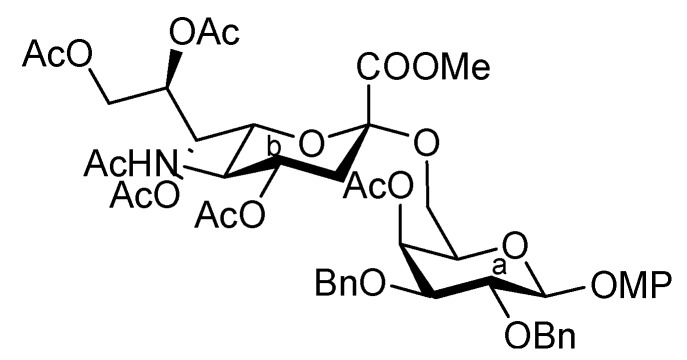



*4-Methoxyphenyl (methyl 5-acetamido-4,7,8,9-tetra-O-acetyl-3,5-dideoxy-D-glycero-α-D-galacto-2-nonulopyranosylonate)-(2→6)-4-O-acetyl-2,3-di-O-benzyl-β-D-galactopyranoside* (**8**). To a solution of **6** (3.77 g, 3.51 μmol) in AcOH/CH_2_Cl_2_ (3:2, 70 mL) was added Zn/Cu couple (18.9 g) at r.t. The reaction mixture was heated to 40 °C and was stirred for 45 min at the same temperature as the reaction was monitored by TLC (4:1 toluene–EtOAc). The precipitate was filtered through Celite and the filtrate was co-evaporated with toluene. The obtained residue was exposed to high vacuum for 6 h. The crude residue was dissolved in pyridine (35 mL) and acetic anhydride (1.32 μL, 14.0 mmol), DMAP (4.3 mg, 35.1 μmol) were then added to the mixture at 0 °C. After stirring for 3 d at r.t as the reaction was monitored by TLC (2:1 toluene–EtOAc), the reaction mixture was evaporated. The residue was diluted with CHCl_3_, washed with 2 M HCl, H_2_O, satd aq NaHCO_3_ and brine, dried over Na_2_SO_4_, and concentrated. The obtained residue was purified by silica gel column chromatography (4:1 toluene–EtOAc) to give **8** (3.35 g, 97%). [α]_D_ −11.3° (c 1.0, CHCl_3_); ^1^H-NMR (500 MHz, CDCl_3_) δ 7.37–7.24 (m, 10 H, 2 Ph), 6.97 (2 d, 4 H, Ar), 5.95 (d, 1 H, *J*_NH,5_ = 6.3 Hz, NH), 5.62 (d, 1 H, *J*_3,4_ = 2.3 Hz, H-4a), 5.41 (m, 1 H, H-8b), 5.32 (d, 1 H, H-7b), 4.96–4.81 (m, 5 H, H-1a, H-4b, 3OCH_2_), 4.68 (d, 1 H, OCH_2_), 4.34 (d, 1 H, H-9b), 4.14–4.05 (m, 3 H, H-5b, H-6b, H-9'b), 3.90–3.69 (m, 11 H, H-2a, H-3a, H-5a, H-6a, H-6'a, 2 OMe), 2.62 (m, 1 H, *J*_gem_ = 11.5 Hz, H-3b*eq*), 2.16–1.87 (m, 19 H, 6 Ac, H-3b*ax*); ^13^C-NMR (100 MHz, CDCl_3_) δ 170.4, 170.2, 170.0, 169.8, 169.7, 169.5, 167.7, 154.9, 151.2, 138.2, 137.5, 128.7, 128.0, 127.9, 127.7, 127.3, 127.2, 118.0, 114.1, 102.2, 98.4, 79.1, 78.3, 75.0, 72.4, 71.8, 71.3, 68.7, 68.3, 67.0, 65.9, 62.7, 62.3, 55.2, 52.5, 48.6, 37.5, 22.7, 20.7, 20.5, 20.4, 20.4, 20.3. *m/z* (MALDI): found [M+Na]^+^ 1004.17, C_49_H_59_NO_20_ calcd for [M+Na]^+^ 1004.35. 



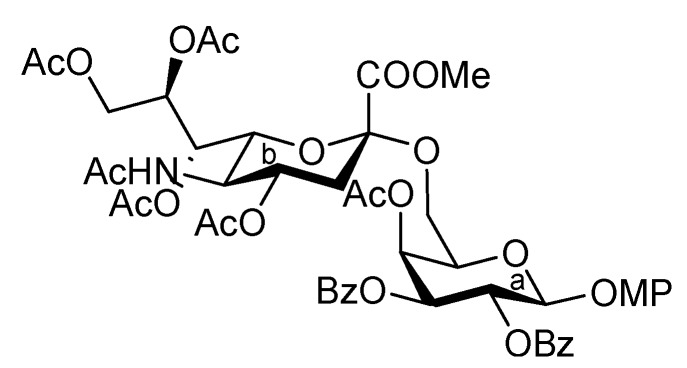



*4-Methoxyphenyl (methyl 5-acetamido-4,7,8,9-tetra-O-acetyl-3,5-dideoxy-D-glycero-α-D-galacto-2-nonulopyranosylonate)-(2→6)-4-O-acetyl-2,3-di-O-benzoyl-β-D-galactopyranoside* (**10**). To a solution of **8** (3.34 g, 3.41 μmol) in 1,4-dioxane (34 mL) was added Pd(OH)_2_/C (3.34 g). After stirring for 45 min at r.t. under a hydrogen atmosphere as the reaction was monitored by TLC (15:1 CHCl_3_–MeOH), the mixture was filtered through Celite. The filtrate was concentrated and the obtained crude residue was roughly purified by silica gel column chromatography. The obtained product was exposed to high vacuum for 24 h. The residue was then dissolved in pyridine (34 mL). Benzoic anhydride (3.09 g, 13.6 mmol) and DMAP (20.8 mg, 0.171 μmol) were added to the mixture at 0 °C. After stirring for 9 h at r.t. as the reaction was monitored by TLC (15:1 CHCl_3_–MeOH), the reaction was quenched by the addition of MeOH at 0 °C. The mixture was co-evaporated with toluene and the residue was then diluted with CHCl_3_, and washed with 2 M HCl, H_2_O, satd aq NaHCO_3_ and brine. The organic layer was subsequently dried over Na_2_SO_4_, and concentrated. The resulting residue was purified by silica gel column chromatography (1:1 toluene–EtOAc) to give **10** (3.22 g, 94%). [α]_D_ +40.3° (c 1.0, CHCl_3_); ^1^H-NMR (500 MHz, CDCl_3_) δ 8.00–7.37 (m, 10 H, 2 Ph), 7.06–6.76 (2 d, Ar), 5.91 (near t, 1 H, *J*_1,2_ = 7.8 Hz, *J*_2,3_ = 10.6 Hz, H-2a), 5.78 (d, 1 H, *J*_3,4_ = 3.4 Hz, H-4a), 5.60 (dd, 1 H, H-3a), 5.50 (m, 1 H, *J*_7,8_ = 6.8 Hz, H-8b), 5.33–5.27 (m, 2 H, H-1a, H-7b), 5.15 (d, 1 H, NH), 4.87 (m, 1 H, *J*_3eq,4_ = 4.6 Hz, H-4b), 4.41 (dd, 1 H, *J*_gem_ = 12.4 Hz, H-9), 4.30 (near t, 1 H, *J*_5,6_ = 6.9 Hz, H-5a), 4.17–4.02 (m, 3 H, H-5b, H-6b, H-9'b), 3.89–3.73 (m, 7 H, 2 OMe, H-6a), 3.58 (near t, 1 H, *J*_gem_ = 10.1 Hz, H-6'a), 2.55 (dd, 1 H, *J*_gem_ = 12.4 Hz, H-3b*eq*), 2.25–1.89 (m, 19 H, 6 Ac, H-3b*ax*); ^13^C-NMR (100 MHz, CDCl_3_) δ 170.5, 170.4, 170.0, 169.7, 169.5, 167.7, 165.1, 165.0, 155.1, 151.0, 133.0, 129.4, 129.3, 129.1, 128.8, 128.1, 128.1, 118.2, 114.1, 100.1, 98.9, 72.5, 71.7, 71.6, 69.4, 68.6, 67.9, 67.0, 62.9, 55.2, 52.7, 48.7, 37.6, 22.8, 20.8, 20.5, 20.4, 20.3. *m/z* (MALDI): found [M+Na]^+^ 1032.21, C_49_H_55_NO_22_ calcd for [M+Na]^+^ 1032.31.



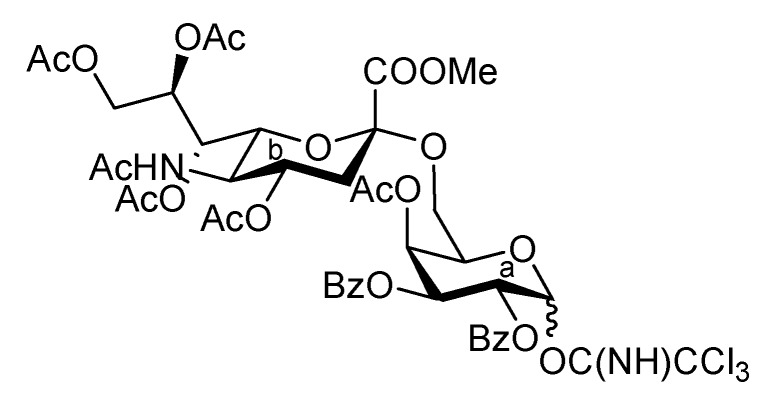



*(Methyl 5-acetamido-4,7,8,9-tetra-O-acetyl-3,5-dideoxy-D-glycero-α-D-galacto-2-nonulopyranosylonate)-(2→6)-4-O-acetyl-2,3-di-O-benzoyl-D-galactopyranosyl trichloroacetimidate* (**12**). To a solution of **10** (3.22 g, 3.19 mmol) in MeCN/PhMe/H_2_O (32 mL, 6:5:3) was added diammonium cerium (IV) nitrate (CAN; 17.5 g, 31.9 mmol) at r.t. The mixture was stirred for 2 h at r.t., as the proceeding of the reaction was monitored by TLC (10:1 CHCl_3_–MeOH). The reaction mixture was diluted with CHCl_3_ and washed with H_2_O, satd aq NaHCO_3_ and brine. The organic layer was subsequently dried over Na_2_SO_4_ and concentrated. The resulting residue was purified by silica gel column chromatography (70:1 CHCl_3_–MeOH) to give **11**. The obtained hemiacetal compound **11** (2.27 g, 2.51 mmol) was dissolved in CH_2_Cl_2_ (50 mL). To the mixture was added CCl_3_CN (2.5 mL, 25.1 mmol), DBU (449 μL, 3.01 mmol) at 0 °C. After stirring for 6 h at 0 °C as the reaction was monitored by TLC (15:1 CHCl_3_–MeOH), the reaction mixture was evaporated. The crude residue was purified by silica gel column chromatography (70:1 CHCl_3_–MeOH) to give **12** (2.10 g, 63% over 2 steps). **12α**: ^1^H-NMR (500 MHz, CDCl_3_) δ 8.61 (s, 1 H, C=NH), 7.95–7.28 (m, 10 H, 2 Ph), 6.80 (d, 1 H, *J*_1,2_ = 3.4 Hz, H-1a), 5.90–5.82 (m, 2 H, H-2a, H-3a), 5.79 (m, 1 H, *J*_3,4_ = 2.8 Hz, H-4a), 5.38–5.30 (m, 3 H, H-7b, H-8b, NHb), 4.87–4.86 (m, 1 H, H-4b), 4.51 (br t, 1 H, H-5a), 4.28 (dd, 1 H, *J*_gem_ = 12.6 Hz, *J*_8,9_ = 2.9 Hz, H-9b), 4.11 (dd, 1 H, *J*_8,9__´_ = 5.9 Hz, H-9'b), 4.06–3.97 (m, 3 H, H-6a, H-5b, H-6b), 3.78 (s, 3 H, COOMe), 3.42 (dd, 1 H, *J*_5,6__'_ = 2.6 Hz, *J*_gem_ = 10.6 Hz, H-6'a), 3.55 (dd, 1 H, *J*_gem_ = 11.6 Hz, *J*_3eq,4_ = 3.6 Hz, H-3b*eq*), 2.35–1.87 (m, 19 H, 6 Ac, H-3b*ax*).



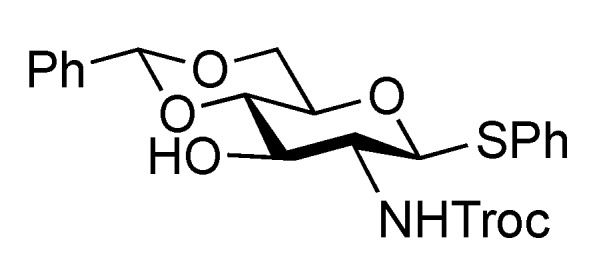



*Phenyl 4,6-O-benzylidene-2-deoxy-1-thio-2-(2,2,2-trichloroethoxycarbamoyl)-β-D-glucopyranoside* (**14**). To a solution of **13** (8.43 g, 14.7 mmol) in MeOH (84 mL) was added NaOMe (28% solution in MeOH, 39.7 mg, 0.735 mmol) at 0 °C. After stirring for 2.5 h at room temperature as the reaction was monitored by TLC (10:1 CHCl_3_–MeOH), the reaction was neutralized with Dowex (H^+^) resin. The resin was filtered through cotton and the filtrate was then evaporated. The residue was exposed to high vacuum for 12 h. The obtained crude mixture was then dissolved in THF/MeCN (1:4, 135 mL). To the mixture were added benzaldehyde dimethyl acetal (4.39 mL, 29.4 mmol) and (±)-camphor-10-sulfonic acid (CSA) (512 mg, 2.21 mmol) at 0 °C. After stirring for 1 h at room temperature as the reaction was monitored by TLC (20:1 CHCl_3_–MeOH), the reaction was quenched by the addition of triethylamine. The reaction mixture was concentrated and then subjected to crystallization from hot acetone/*n*-hexane to give **14** (6.27 g, 80%) as a white crystal. The physical data of **14** was identical to those reported in the literature [27].



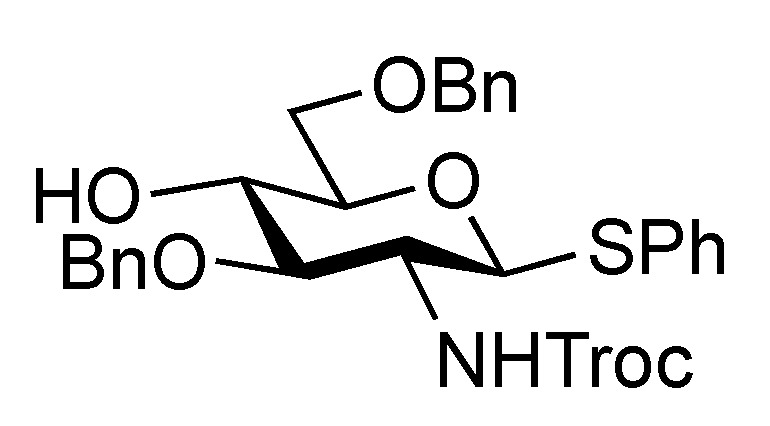



*Phenyl 3,6-di-O-benzyl-2-deoxy-1-thio-2-(2,2,2-trichloroethoxycarbamoyl)-β-D-glucopyranoside* (**15**). To a solution of **14** (4.89 g, 9.15 mmol) in THF/toluene (1:4, 9.0 mL) was added TESOTf (4.1 mL, 18.3 mmol) at −20 °C. After stirring for 45 min at −20 °C, benzaldehyde (4.7 mL, 45.7 mmol) and triethylsilane (2.2 mL, 13.7 mmol) were added to the mixture. After stirring for 2 h at −20 °C as the reaction was monitored by TLC (1:4 EtOAc–toluene), the reaction was quenched by satd aq Na_2_CO_3_. Dilution of the mixture with EtOAc provided a solution, which was then washed with satd aq Na_2_CO_3_ and brine. The organic layer was subsequently dried over Na_2_SO_4_ and concentrated. The obtained residue was exposed to high vacuum for 24 h. The resulting residue was dissolved in CH_2_Cl_2_ (183 mL) and cooled to 0 °C. BF_3_·OEt_2_ (4.7 mL, 18.3 mmol) and triethylsilane (14.6 mL, 91.5 mmol) were added to the solution at 0 °C and the mixture was then stirred for 1 h at 0 °C as the reaction was monitored by TLC (1:4 EtOAc–toluene). The reaction was quenched by the addition of satd aq Na_2_CO_3_ at 0 °C and then diluted with CHCl_3_, and washed with satd aq Na_2_CO_3_ and brine. The organic layer was subsequently dried over Na_2_SO_4_ and concentrated. The resulting residue was purified by silica gel column chromatography (200:1 CHCl_3_–MeOH) to give **15** (4.50 g, 78%). [α]_D_ −24.2° (c 0.3, CHCl_3_); ^1^H-NMR (400 MHz, CDCl_3_) δ 7.49–7.21 (m, 15 H, 3 Ph), 5.17 (d, 1 H, *J*_2,NH_ = 8.2 Hz, NH), 4.91 (d, 1 H, , *J*_1,2_ = 10.1 Hz, H-1), 4.75 (s, 4 H, 2 OCH_2_), 4.56 (2 d, 2 H, OCH_2_), 3.77–3.67 (m, 4 H, H-3, H-5, H-6, H-6'), 3.52–3.42 (m, 2 H, H-2, H-4), 2.85 (s, 1 H, OH); ^13^C-NMR (100 MHz, CDCl_3_) δ 153.8, 137.9, 137.7, 132.7, 132.3, 128.9, 128.5, 128.4, 128.1, 128.0, 127.8, 127.7, 95.4, 86.0, 81.9, 78.0, 74.5, 74.4, 73.7, 72.6, 70.4, 56.0. *m/z* (MALDI): found [M+Na]^+^ 648.05, C_29_H_30_Cl_3_NO_6_S calcd for [M+Na]^+^ 648.08.



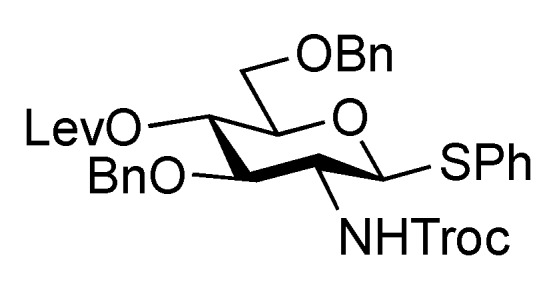



*Phenyl 3,6-di-O-benzyl -2-deoxy-4-O-levulinoyl-1-thio-2-(2,2,2-trichloroethoxycarbamoyl)-β-D-glucopyranoside* (**16**). To a solution of **15** (200 mg, 0.314 mmol) in CH_2_Cl_2_ (3.1 mL) were added levulinic acid (55 mg, 0.472 mmol), EDC·HCl (90 mg, 0.472 mmol), and DMAP (4.2 mg, 3.44 μmol). After stirring for 4 h at r.t. as the reaction was monitored by TLC (2:3 EtOAc–*n*-hexane), the reaction mixture was diluted with CHCl_3_, and washed with 2 M HCl and brine. The organic layer was subsequently dried over Na_2_SO_4_, and concentrated, and the obtained residue was purified by silica gel column chromatography (1:4 EtOAc–*n*-hexane) to give **16** (215 mg, 93%). [α]_D_ +3.7° (c 0.9, CHCl_3_); ^1^H-NMR (600 MHz, acetone-*d*_6_) δ 7.53–7.24 (m, 16 H, 3 Ph, NH), 5.06 (d, 1 H, *J*_1,2_ = 10.3 Hz, H-1), 5.03 (t, 1 H, *J*_3,4_ = 9.6 Hz, *J*_4,5_ = 9.7 Hz, H-4), 4.83 (2 d, 2 H, *J*_gem_ = 12.4 Hz, OCH_2_), 4.72 (2 d, 2 H, *J*_gem_ = 11.0 Hz, OCH_2_), 4.52 (2 d, 2 H, *J*_gem_ = 11.7 Hz, OCH_2_), 3.99 (t, 1 H, *J*_2,3_ = 9.7 Hz, H-3), 3.79–3.75 (m, 2 H, H-2, H-5), 3.65 (dd, 1 H, *J*_gem_ = 11.0 Hz, *J*_5,6_ = 2.8 Hz, H-6), 3.65 (dd, 1 H, *J*_5,6'_ = 6.9 Hz, H-6'), 2.73–2.40 (m, 4 H, CH_2_CH_2_C(=O)O), 2.09 (s, 3 H, C(=O)CH_3_); ^13^C-NMR (150 MHz, CDCl_3_) δ 166.1, 153.7, 137.6, 137.5, 132.6, 132.1, 129.0, 128.5, 128.3, 128.0, 128.0, 127.9, 127.7, 95.4, 85.2, 79.0, 76.8, 74.5, 74.4, 73.6, 73.2, 69.5, 56.7, 40.5. *m/z* (MALDI): found [M+Na]^+^ 746.09, C_34_H_36_Cl_3_NO_8_S calcd for [M+Na]^+^ 746.11.



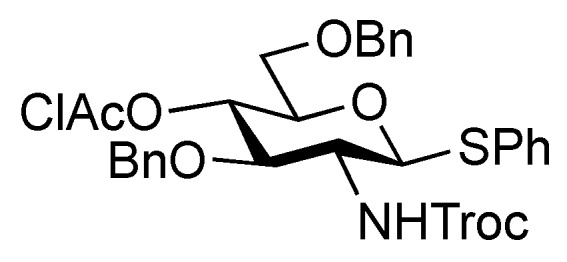



*Phenyl 3,6-di-O-benzyl-4-O-chloroacetyl-2-deoxy-1-thio-2-(2,2,2-trichloroethoxycarbamoyl)-β-D-glucopyranoside* (**17**). To a solution of **15** (200 mg, 0.314 mmol) in THF (3.1 mL) were added monochloroacetic anhydride (81 mg, 0.472 mmol) and DMAP (0.5 mg, 4.09 μmol) at 0 °C. After stirring for 7 h at r.t. as the reaction was monitored by TLC (2:3 EtOAc–*n*-hexane), THF was evaporated and the residue was then diluted with CHCl_3_, and washed with 2 M HCl, H_2_O, satd aq NaHCO_3_, and brine. The organic layer was subsequently dried over Na_2_SO_4_, and concentrated. The residue was purified by silica gel column chromatography (1:8 EtOAc–*n*-hexane) to give **17** (200 mg, 90%). [α]_D_ −2.6° (c 1.0, CHCl_3_); ^1^H-NMR (600 MHz, CDCl_3_) δ 7.51–7.21 (m, 15 H, 3 Ph), 5.37 (d, 1 H, *J*_NH,2_ = 8.2 Hz, NH), 5.12 (d, 1 H, *J*_1,2_ = 10.3 Hz, H-1), 5.07 (near t, 1 H, *J*_3,4_ = 8.9 Hz, *J*_4,5_ = 9.6 Hz, H-4), 4.75 (2 d, 2 H, *J*_gem_ = 11.8 Hz, OCH_2_), 4.61 (2 d, 2 H, *J*_gem_ =11.7 Hz, OCH_2_), 4.48 (m, 2 H, *J*_gem_ = 11.7 Hz, OCH_2_), 4.11 (near t, 1 H, *J*_2,3_ = 9.6 Hz, *J*_3,4_ = 8.9 Hz, H-3), 3.68–3.55 (m, 5 H, H-5, H-6, H-6', CH_2_Cl), 3.36 (br dd, 1 H, *J*_2,3_ = 9.6 Hz, H-2); ^13^C-NMR (125 MHz, CDCl_3_) δ 166.1, 153.7, 137.7, 137.6, 132.8, 132.0, 129.1, 128.5, 128.4, 128.2, 128.0, 127.9, 127.8, 95.4, 85.2, 79.0, 77.6, 74.7, 74.5, 73.7, 73.4, 69.7, 56.9, 40.5. *m/z* (MALDI): found [M+Na]^+^ 724.05, C_31_H_31_Cl_4_NO_7_S calcd for [M+Na]^+^ 724.05. 



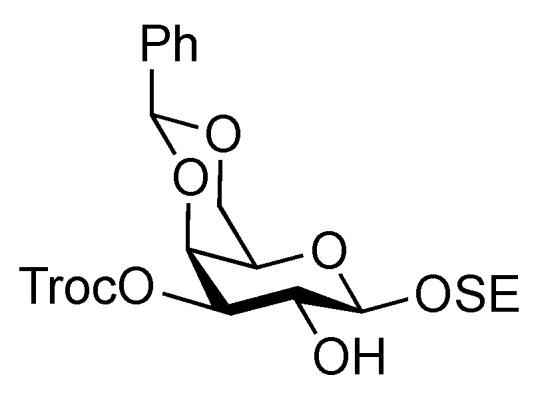



*2-(Trimethylsilyl)ethyl 4,6-O-benzylidene-3-O-(2,2,2-trichloroethoxycarbonyl)-β-D-galactopyranoside* (**19**). To a solution of **18** (49.5 mg, 0.134 mmol) in pyridine/CH_2_Cl_2_ (1:4, 1.3 mL) was added trichloroethyl chloroformate (20 μL, 0.148 mmol) at −40 °C. After stirring for 3 h at the same temperature as the reaction was monitored by TLC (30:1 CHCl_3_–MeOH), solvents were removed by co-evaporation with toluene, and then the residue was diluted with CHCl_3_, washed with 2 M HCl, H_2_O, satd aq NaHCO_3_, and brine. The organic layer was subsequently dried over Na_2_SO_4_, and concentrated. The resulting residue was purified by silica gel column chromatography (1:5 EtOAc–*n*-hexane) to give **19** (59.1 mg, 81%). [α]_D_ +41.7° (c 1.0, CHCl_3_); ^1^H-NMR (400 MHz, CDCl_3_) δ 7.48–7.30 (m, 5 H, Ph), 5.49 (s, 1 H, PhC*H*<), 4.82–4.72 (m, 3 H, H-3, OCH_2_CCl_3_), 4.45 (d, 1 H, *J*_3,4_ = 3.7 Hz, H-4), 4.34 (d, 1 H, *J*_1,2_ = 7.7 Hz, H-1), 4.33 (d, 1 H, *J*_gem_ = 13.8 Hz, H-6), 4.08–4.01 (m, 3 H, H-2, H-6', OC*H_2_*CH_2_SiMe_3_), 3.60–3.53 (m, 1 H, OC*H_2_*CH_2_SiMe_3_), 3.48 (s, 1 H, H-5), 2.51 (br s, 1 H, OH), 1.03–0.97 (m, 2 H, OCH_2_C*H_2_*SiMe_3_), 0.00 (s, 9 H, OCH_2_CH_2_Si*Me_3_*); ^13^C-NMR (100 MHz, CDCl_3_) δ 153.6, 137.3, 129.0, 128.1, 126.2, 102.3, 100.9, 94.2, 78.2, 77.2, 76.9, 73.0, 69.0, 68.5, 67.5, 66.2, 18.1, −1.4. *m/z* (MALDI): found [M+Na]^+^ 565.11, C_16_H_16_Cl_3_O_7_ calcd for [M+Na]^+^ 565.06.



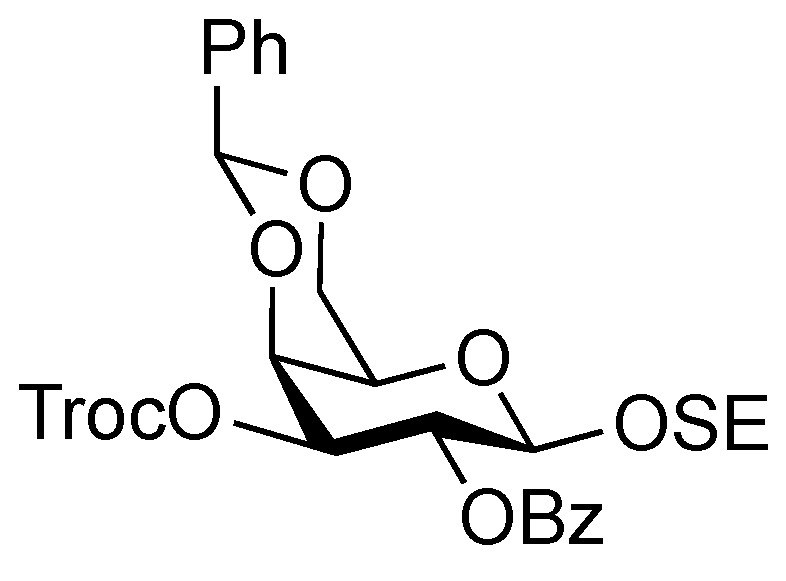



*2-(Trimethylsilyl)ethyl 2-O-benzoyl-4,6-O-benzylidene-3-O-(2,2,2-trichloroethoxycarbonyl)-β-D-galactopyranoside* (**20**). To a solution of **19** (2.39 g, 4.39 mmol) in THF (1.3 mL) were added benzoic anhydride (1.49 g, 6.59 mmol) and DMAP (268 mg, 2.20 μmol) at 0 °C. After stirring for 8.5 h at r.t. as the reaction was monitored by TLC (30:1 CHCl_3_–MeOH), THF was evaporated and the residue was then diluted with CHCl_3_, washed with 2 M HCl, H_2_O, satd aq NaHCO_3_, and brine. The organic layer was subsequently dried over Na_2_SO_4_, concentrated. The residue was purified by silica gel column chromatography (20:1 toluene–EtOAc) to give **20** (2.90 g, quant.). [α]_D_ +38.8° (c 1.2, CHCl_3_); ^1^H-NMR (400 MHz, CDCl_3_) δ 8.11–7.43 (m, 10 H, 2 Ph), 5.79–7.75 (near t, 1 H, *J*_2,3_ = 10.6 Hz, *J*_1,2_ = 7.8 Hz, H-2), 5.64 (s, 1 H, PhC*H*<), 5.22 (dd, 1 H, *J*_3,4_ = 3.7 Hz, H-3), 4.81–4.78 (2 d, 2 H, OCH_2_CCl_3_, H-1), 4.68 (d, 1 H, *J*_gem_ = 11.9 Hz, OCH_2_CCl_3_), 4.58 (d, 1 H, H-4), 4.67–4.50 (dd, 1 H, *J*_gem_ = 12.4 Hz, *J*_5,6_ = 1.8 Hz, H-6), 4.23–4.19 (dd, 1 H, *J*_5,6´_ = 1.8 Hz, H-6'), 4.15–4.08 (m, 1 H, OC*H_2_*CH_2_SiMe_3_), 3.68–3.62 (m, 2 H, H-5, OC*H_2_*CH_2_SiMe_3_), 1.03–0.91 (m, 2 H, OCH_2_C*H_2_*SiMe_3_), 0.00 (s, 9 H, OCH_2_CH_2_Si*Me_3_*); ^13^C-NMR (100 MHz, CDCl_3_) δ 164.8, 153.7, 137.2, 133.1, 129.8, 129.7, 129.1, 128.3, 128.2, 126.4, 101.1, 100.5, 94.1, 76.8, 76.6, 73.4, 69.2, 68.9, 67.0, 66.2, 17.9, −1.5. *m/z* (MALDI): found [M+Na]^+^ 669.09, C_28_H_33_Cl_3_O_9_Si calcd for [M+Na]^+^ 669.09.



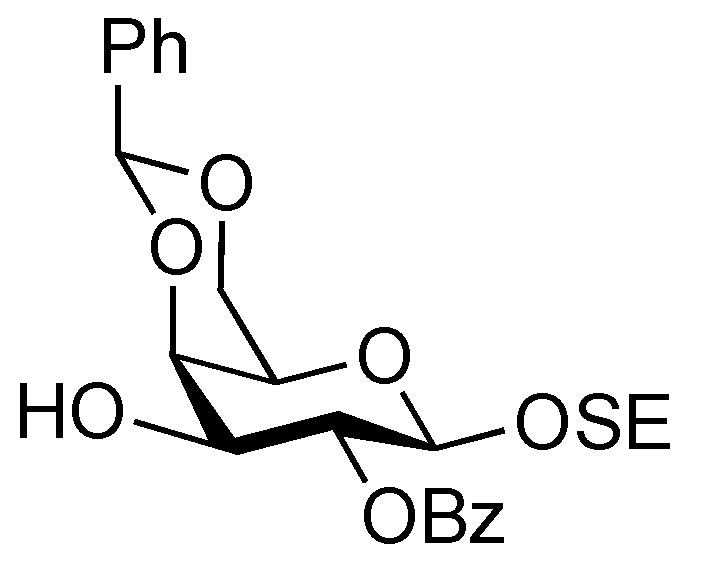



*2-(Trimethylsilyl)ethyl 2-O-benzoyl-4,6-O-benzylidene-β-D-galactopyranoside* (**21**). To a solution of **20** (2.18 g, 3.36 μmol) in AcOH/CH_2_Cl_2_ (3:2, 33.6 mL) was added Zn/Cu couple (6.00 g) at r.t. The reaction mixture was stirred for 1 h at r.t. as the reaction was monitored by TLC (30:1 CHCl_3_–MeOH). The precipitate was filtered through Celite and the filtrate was co-evaporated with toluene. The residue was diluted with CHCl_3_ and washed with satd aq NaHCO_3_ and brine, dried over Na_2_SO_4_, and concentrated. The obtained residue was purified by silica gel column chromatography (4:1 EtOAc–*n*-hexane) to give **21** (1.33 g, 84%). [α]_D_ −3.8° (c 1.1, CHCl_3_); ^1^H-NMR (400 MHz, CDCl_3_) δ 8.15–7.45 (m, 10 H, 2 Ph), 5.65 (s, 1 H, PhC*H*<), 5.43 (near t, 1 H, *J*_2,3_ = 10.3 Hz, *J*_1,2_ = 8.3 Hz, H-2), 4.68 (d, 1 H, *J*_1,2_ = 8.3 Hz, H-1), 4.45 (d, 1 H, *J*_gem_ = 12.3 Hz, H-6), 4.33 (d, 1 H, *J*_3,4_ = 4.2 Hz, H-4), 4.18 (dd, 1 H, *J*_gem_ = 12.4 Hz, H-6'), 4.12 (m, 1 H, OC*H_2_*CH_2_SiMe_3_), 3.97 (m, 1 H, *J*_3,OH_ = 11.0 Hz, *J*_2,3_ = 10.3 Hz, H-3), 3.68–3.60 (m, 2 H, H-5, OC*H_2_*CH_2_SiMe_3_), 2.72 (d, 1 H, OH), 0.98 (m, 2 H, CH_2_C*H_2_*SiMe_3_), 0.00 (s, 3 H, CH_2_CH_2_Si*Me_3_*); ^13^C-NMR (100 MHz, CDCl_3_) δ 166.2, 137.4, 132.9, 130.0, 129.8, 129.2, 128.2, 126.4, 101.4, 100.3, 75.6, 72.9, 71.9, 69.0, 67.0, 66.5, 17.9, −1.5. *m/z* (MALDI): found [M+Na]^+^ 495.12, C_25_H_32_O_7_Si calcd for [M+Na]^+^ 495.18. 



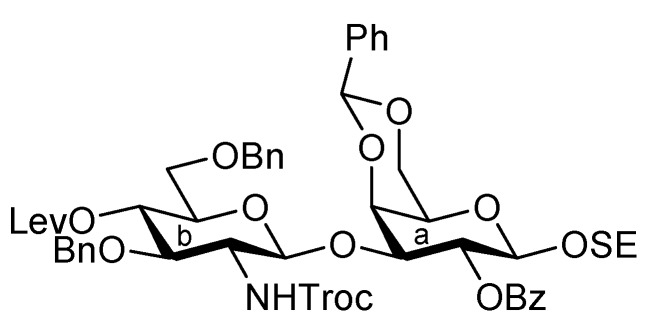



*2-(Trimethylsilyl)ethyl 3,6-di-O-benzyl-2-deoxy-4-O-levulinoyl-2-(2,2,2-trichloroethoxycarbamoyl)-β-D-glucopyranosyl-(1→3)-2-O-benzoyl-4,6-O-benzylidene-β-D-galactopyranoside* (**22**). To a mixture of **16** (230 mg, 0.317 mmol) and **21** (100 mg, 0.212 mmol) in CH_2_Cl_2_ (3.2 mL) was added 4 Å molecular sieves AW-300 (400 mg) at r.t. After stirring for 1 h, the mixture was cooled to 0 °C. NIS (143 mg, 0.636 mmol) and TfOH (2.8 μL, 31.7 μmol) were then added to the mixture at 0 °C. After stirring for 2 h at the same temperature as the reaction was monitored by TLC (1:4 EtOAc–toluene), the reaction was quenched by the addition of triethylamine. The solution was diluted with CHCl_3_ and filtered through Celite. The filtrate was then washed with satd aq Na_2_S_2_O_3_ and brine. The organic layer was subsequently dried over Na_2_SO_4_, and concentrated. The resulting residue was purified by silica gel column chromatography (1:4 EtOAc–toluene, then 200:1 CHCl_3_–MeOH) to give **22** (186 mg, 81%). [α]_D_ +17.4° (c 0.8, CHCl_3_); ^1^H-NMR (600 MHz, CDCl_3_) δ 8.07–7.13 (m, 20 H, 4 Ph), 5.55 (near t, 1 H, *J*_2,3_ = 10.3 Hz, *J*_1,2_ = 7.6 Hz, H-2a), 5.49 (s, 1 H, PhCH<), 5.26 (d, 1 H, *J*_NH,2_ = 6.2 Hz, NH), 5.08 (d, 1 H, *J*_1,2_ = 7.6 Hz, H-1b), 4.92 (near t, 1 H, *J*_3,4_ = 9.9 Hz, *J*_4,5_ = 9.4 Hz, H-4b), 4.53 (d, 1 H, H-1a), 4.55–4.51 (m, 2 H, 2 OCH_2_), 4.46–4.41 (m, 3 H, 3 OCH_2_), 4.40 (d, 1 H, *J*_3,4_ = 3.4 Hz, H-4a), 4.26 (d, 1 H, *J*_gem_ = 12.4 Hz, H-6'a), 4.15 (t, 1 H, *J*_3,4_ = 9.9 Hz, H-3b), 4.00–3.95 (m, 2 H, H-3a, OCH_2_CH_2_SiMe_3_), 3.83 (d, 1 H, *J*_gem_ = 12.4 Hz, H-6a), 3.66 (br m, 1 H, H-5b), 3.55 (m, 3 H, H-6b, H-6'b, OCH_2_CH_2_SiMe_3_), 3.35 (s, 1 H, H-5a), 3.28 (br d, 1 H, OCH_2_), 3.17 (br dd, 1 H, H-2b), 2.61–2.36 (m, 4 H, CH_2_CH_2_C(=O)O), 2.10 (s, 3 H, C(=O)CH_3_), 0.86–0.76 (m, 2 H, CH_2_CH_2_SiMe_3_), −0.12 (m, 9 H, CH_2_CH_2_SiMe_3_); ^13^C-NMR (125 MHz, CDCl_3_) δ 206.2, 171.6, 164.9, 153.4, 138.1, 138.0, 137.8, 133.0, 130.2, 129.9, 128.9, 128.4, 128.3, 128.3, 128.1, 127.7, 127.6, 127.6, 127.5, 126.5, 101.1, 100.8, 100.2, 95.4, 78.7, 77.5, 76.2, 74.2, 73.4, 73.3, 73.2, 71.6, 70.5, 69.9, 68.8, 66.7, 66.6, 58.2, 37.7, 29.7, 27.8, 17.9, −1.6. *m/z* (MALDI): found [M+Na]^+^ 1108.51, C_53_H_62_Cl_3_NO_15_Si calcd for [M+Na]^+^ 1108.29.



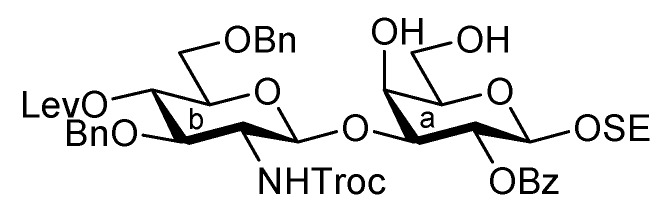



*2-(Trimethylsilyl)ethyl 3,6-di-O-benzyl-2-deoxy-4-O-levulinoyl-2-(2,2,2-trichloroethoxycarbamoyl)-β-D-glucopyranosyl-(1→3)-2-O-benzoyl-β-D-galactopyranoside* (**23**). Compound **22** (42 mg, 38.3 μmol) was dissolved in 80% AcOH aq (1.5 mL) and the solution was stirred for 4 h at 50 °C as the reaction was monitored by TLC (15:1 CHCl_3_–MeOH). After the reaction mixture was co-evaporated with toluene, the crude residue was purified by silica gel column chromatography (50:1 CHCl_3_–MeOH) to give **23** (38 mg, 99%). [α]_D_ −1.43° (c 0.7, CHCl_3_); ^1^H-NMR (400 MHz, acetone-d_6_) δ 8.34–7.32 (m, 15 H, 3 Ph), 7.03 (d, 1 H, *J*_NH,2_ = 9.2 Hz, NH), 5.60 (near t, 1 H, *J*_2,3_ = 9.7 Hz, *J*_1,2_ = 8.0 Hz, H-2a), 5.13 (d, 1 H, *J*_1,2_ = 8.6 Hz, H-1b), 5.09 (near t, *J*_3,4_ = 9.9 Hz, *J*_4,5_ = 8.3 Hz, H-4b), 4.77–4.64 (m, 5 H, H-1a, 4 OCH_2_), 4.48 (d, 1 H, *J*_3,4_ = 3.4 Hz, H-4a), 4.51 (d, 1 H, OCH_2_), 4.23 (dd, 1 H, H-3a), 4.13–4.08 (m, 2 H, H-3b, OCH_2_), 3.96–3.63 (m, 9 H, 2 OCH_2_, H-2b, H-5b, H-6b, H-6'b, H-5a, H-6a, H-6'a), 2.98 (s, 2 H, 2 OH), 2.80–2.49 (m, 4 H, CH_2_CH_2_C(=O)O), 2.20 (s, 3 H, C(=O)CH_3_), 1.01–0.83 (m, 2 H, CH_2_CH_2_SiMe_3_), 0.00 (s, 9 H, CH_2_CH_2_SiMe_3_); ^13^C-NMR (100 MHz, acetone-d_6_) δ 206.5, 172.5, 165.6, 154.8, 139.5, 133.7, 131.7, 130.7, 129.2, 129.0, 128.8, 128.4, 128.3, 128.2, 128.0, 102.7, 101.7, 96.8, 82.4, 80.2, 75.9, 74.6, 74.1, 74.0, 73.9, 72.1, 72.0, 70.4, 69.5, 67.0, 62.6, 58.3, 38.1, 29.6, 28.6, 18.6, −1.4. *m/z* (MALDI): found [M+Na]^+^ 1020.26, C_46_H_58_Cl_3_NO_15_Si calcd for [M+Na]^+^ 1020.25.



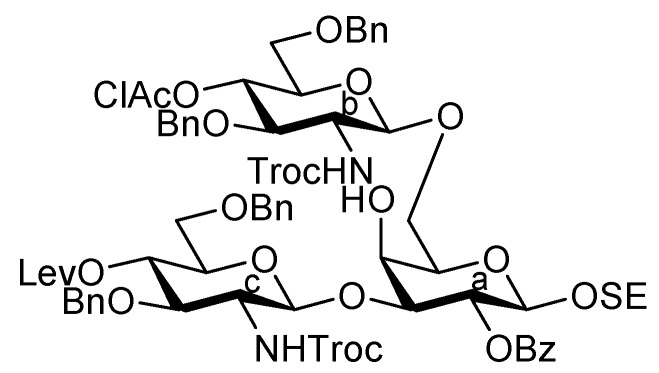



*2-(Trimethylsilyl)ethyl 3,6-di-O-benzyl-2-deoxy-4-O-levulinoyl-2-(2,2,2-trichloroethoxycarbamoyl)-β-D-glucopyranosyl-(1→3)-[3,6-di-O-benzyl-4-O-chloroacetyl-2-deoxy-2-(2,2,2-trichloroethoxycarbamoyl)-β-D-glucopyranosyl-(1→6)]-2-O-benzoyl-β-D-galactopyranoside* (**24**). To a mixture of **17** (694 mg, 0.987 mmol) and **23** (658 mg, 0.658 mmol) in CH_2_Cl_2_ (6.6 mL) was added 4 Å molecular sieves (1.40 g) at r.t. After stirring for 1 h, the mixture was cooled to 0 °C. NIS (296 mg, 1.32 mmol) and TfOH (8.7 μL, 98.7 μmol) were then added to the mixture at 0 °C. After stirring for 2 h at r.t. as the reaction was monitored by TLC (30:1 CHCl_3_–MeOH), the reaction was quenched by the addition of triethylamine. The solution was diluted with CHCl_3_ and filtered through Celite. The filtrate was then washed with satd aq Na_2_S_2_O_3_ and brine. The organic layer was subsequently dried over Na_2_SO_4_, and concentrated. The residue was purified by silica gel column chromatography (100:1 CHCl_3_–MeOH) to give **24** (511 mg, 49%). [α]_D_ −19.4° (c 1.8, CHCl_3_); ^1^H-NMR (400 MHz, DMSO-d_6_) δ 8.20–7.32 (m, 27 H, 5 Ph, NHb, NHc), 5.38 (t, 1 H, *J*_2,3_ = 8.3 Hz, *J*_1,2_ = 8.7 Hz, H-2a), 5.14 (t, 1 H, *J*_4,5_ = 9.6 Hz, H-4c), 5.06 (d, 1 H, *J*_gem_ = 12.4 Hz, OCH_2_), 5.01 (t, 1 H, *J*_3,4_ = 9.6 Hz, *J*_4,5_ = 9.1 Hz, H-4b), 4.90 (d, 1 H, *J*_1,2_ = 8.2 Hz, H-1c), 4.75 (d, 1 H, *J*_1,2_ = 8.3 Hz, H-1b), 4.66 (d, 1 H, H-1a), 4.91–4.61 (m, 10 H, OH, 9 OCH_2_), 4.49 (2 d, 2 H, OCH_2_), 4.24 (br s, 1 H, H-4a), 4.04–3.52 (m, 18 H, H-3a, H-5a, H-6a, H-6'a, H-2b, H-3b, H-5b, H-6b, H-6'b, H-2c, H-3c, H-5c, H-6c, H-6'c, OCH_2_CH_2_SiMe_3_, CH_2_Cl), 2.83–2.80 (m, 2 H, CH_2_CH_2_C(=O)O), 2.56 (m, 2 H, CH_2_CH_2_C(=O)O), 2.26 (s, 3 H, C(=O)CH_3_), 0.93–0.78 (m, 2 H, CH_2_CH_2_SiMe_3_), 0.00 (s, 9 H, CH_2_CH_2_SiMe_3_); ^13^C-NMR (100 MHz, DMSO-d_6_) δ 206.8, 171.5, 166.5, 164.7, 154.4, 153.8, 138.4, 138.3, 138.2, 133.2, 130.3, 129.6, 128.6, 128.4, 128.3, 128.2, 127.7, 127.7, 127.6, 101.9, 101.1, 100.2, 96.4, 96.0, 80.7, 79.4, 78.9, 73.7, 73.6, 73.3, 72.8, 72.7, 72.6, 72.5, 72.2, 72.0, 70.9, 70.7, 69.6, 69.0, 68.7, 68.6, 66.1, 57.0, 41.1, 37.4, 29.7, 27.8, 17.6, −1.3. *m/z* (MALDI): found [M+Na]^+^ 1611.27, C_71_H_83_Cl_7_N_2_O_22_Si calcd for [M+Na]^+^ 1611.29.



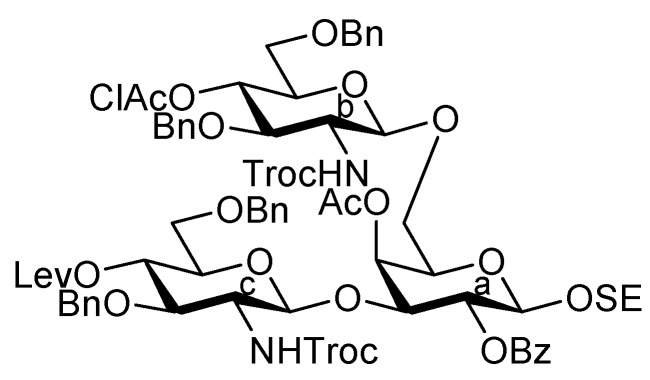



*2-(Trimethylsilyl)ethyl 3,6-di-O-benzyl-2-deoxy-4-O-levulinoyl-2-(2,2,2-trichloroethoxycarbamoyl)-β-D-glucopyranosyl-(1→3)-[3,6-di-O-benzyl-4-O-chloroacetyl-2-deoxy-2-(2,2,2-trichloroethoxycarbamoyl)-β-D-glucopyranosyl-(1→6)]-4-O-acetyl-2-O-benzoyl-β-D-galactopyranoside* (**27**). To a solution of **24** (759 mg, 0.476 mmol) in THF (4.8 mL) were added acetic anhydride (94 μL, 0.956 mmol) and DMAP (5.8 mg, 47.6 μmol) at 0 °C. The reaction mixture was stirred for 1.5 h at r.t. as the reaction was monitored by TLC (15:1 CHCl_3_–MeOH). After THF was evaporated, the obtained crude residue was purified by silica gel column chromatography (100:1 CHCl_3_–MeOH) to give **27** (745 mg, 96%). [α]_D_ −5.5° (c 1.8, CHCl_3_); ^1^H-NMR (400 MHz, DMSO-d_6_) δ 8.20–7.32 (m, 27 H, 5 Ph, NHb, NHc), 5.58 (br s, 1 H, H-4a), 5.34 (near t, 1 H, *J*_2,3_ = 9.7 Hz, *J*_1,2_ = 8.2 Hz, H-2a), 5.16–5.07 (m, 3 H, H-4c, H-4b, OCH_2_), 4.86–4.61 (m, 10 H, H-1b, H-1c, H-1a, 7 OCH_2_), 4.58–4.39 (m, 3 H, 3 OCH_2_), 4.31 (br dd, 1 H, H-3a), 4.18 (br dd, 1 H, H-6a), 4.06–3.48 (m, 17 H, H-5a, H-6'a, H-2b, H-3b, H-5b, H-6b, H-6'b, H-2c, H-3c, H-5c, H-6c, H-6'c, OCH_2_, OCH_2_CH_2_SiMe_3_, CH_2_Cl), 2.80 (m, 2 H, CH_2_CH_2_C(=O)O), 2.54 (m, 2 H, CH_2_CH_2_C(=O)O), 2.25–2.22 (2 s, 6 H, 2 C(=O)CH_3_), 0.96–0.79 (m, 2 H, CH_2_CH_2_SiMe_3_), 0.00 (s, 9 H, CH_2_CH_2_SiMe_3_); ^13^C-NMR (100 MHz, DMSO-d_6_) δ 206.7, 179.5, 171.3, 170.0, 166.5, 164.5, 154.4, 153.8, 138.5, 138.3, 138.3, 138.2, 133.3, 130.1, 129.6, 128.7, 128.4, 128.3, 128.3, 128.1, 127.7, 127.6, 127.6, 127.5, 127.2, 101.3, 100.9, 100.0, 96.3, 96.0, 79.3, 79.0, 77.6, 73.6, 73.1, 72.7, 72.4, 72.3, 72.0, 71.1, 70.3, 70.2, 69.0, 68.8, 68.4, 66.4, 57.0, 41.1, 37.4, 29.7, 27.8, 20.8, 17.6, −1.3. *m/z* (MALDI): found [M+Na]^+^ 1653.57, C_73_H_85_Cl_7_N_2_O_23_Si calcd for [M+Na]^+^ 1653.30.



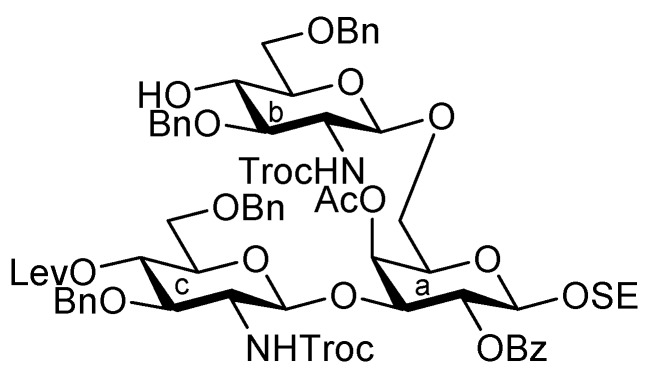



*2-(Trimethylsilyl)ethyl 3,6-di-O-benzyl-2-deoxy-4-O-levulinoyl-2-(2,2,2-trichloroethoxycarbamoyl)-β-D-glucopyranosyl-(1→3)-[3,6-di-O-benzyl-2-deoxy-2-(2,2,2-trichloroethoxycarbamoyl)-β-D-glucopyranosyl-(1→6)]-4-O-acetyl-2-O-benzoyl-β-D-galactopyranoside* (**28**). To a solution of **27** (712 mg, 0.435 mmol) in EtOH (4.4 mL) was added DABCO (732 mg, 6.52 mmol) at 0 °C. After stirring for 1 h at 50 °C as the reaction was monitored by TLC (30:1 CHCl_3_–MeOH), EtOH was evaporated. The obtained crude residue was purified by silica gel column chromatography (100:1 CHCl_3_–MeOH) to give **28** (665 mg, 98%). [α]_D_ −1.8° (c 1.5, CHCl_3_); ^1^H-NMR (500 MHz, CDCl_3_) δ 8.19–7.27 (m, 25 H, 5 Ph), 5.63 (d, 1 H, *J*_3,4_ = 3.4 Hz, H-4a), 5.52 (near t, 1 H, *J*_2,3_ = 10.3 Hz, *J*_1,2_ = 8.0 Hz, H-2a), 5.32 (br d, 1 H, NHb), 5.18 (d, 1 H, *J*_NH,2_ = 6.9 Hz, NHc), 5.07 (d, 1 H, *J*_1,2_ = 9.2 Hz, H-1c), 5.05 (near t, 1 H, *J*_3,4_ = 9.2 Hz, H-4c), 4.85 (d, 1 H, *J*_1,2_ = 8.6 Hz, H-1b), 4.91–4.80 (m, 3 H, 3 OCH_2_), 4.71 (d, 1 H, OCH_2_), 4.63 (d, 1 H, H-1a), 4.69–4.59 (m, 6 H, 6 OCH_2_), 4.50 (d, 1 H, OCH_2_), 4.16 (br t, 1 H, *J*_2,3_ = 10.3 Hz, H-3c), 4.09 (m, 1 H, OCH_2_CH_2_SiMe_3_), 4.07 (dd, 1 H, H-3a), 4.01 (dd, 1 H, *J*_5,6_ = 4.1 Hz, *J*_gem_ = 12.8 Hz, H-6a), 3.89–3.56 (m, 11 H, H-5a, H-6'a, H-3b, H-5b, H-6b, H-6'b, H-5c, H-6c, H-6'c, OCH_2_, OCH_2_CH_2_SiMe_3_), 3.44 (br dd, 1 H, H-2b), 3.23 (br dd, 1 H, H-2c), 2.96 (s, 1 H, OH), 2.72–2.37 (m, 4 H, CH_2_CH_2_C(=O)O), 2.22 (m, 6 H, 2 C(=O)CH_3_), 1.01–0.86 (m, 2 H, CH_2_CH_2_SiMe_3_), 0.00 (s, 9 H, CH_2_CH_2_SiMe_3_); ^13^C-NMR (100 MHz, CDCl_3_) δ 206.1, 171.4, 169.9, 164.8, 154.0, 153.3, 138.2, 138.1, 137.7, 137.5, 133.2, 129.9, 128.5, 128.4, 128.3, 128.0, 127.9, 127.8, 127.7, 127.6, 127.6, 100.7, 100.2, 95.6, 95.4, 80.5, 77.5, 74.3, 74.3, 73.9, 73.7, 73.4, 73.4, 73.2, 73.1, 71.5, 71.4, 70.5, 70.1, 69.6, 67.4, 57.9, 57.3, 37.6, 29.6, 27.8, 20.8, 17.9, −1.5. *m/z* (MALDI): found [M+Na]^+^ 1577.28, C_71_H_84_Cl_6_N_2_O_22_Si calcd for [M+Na]^+^ 1577.33.



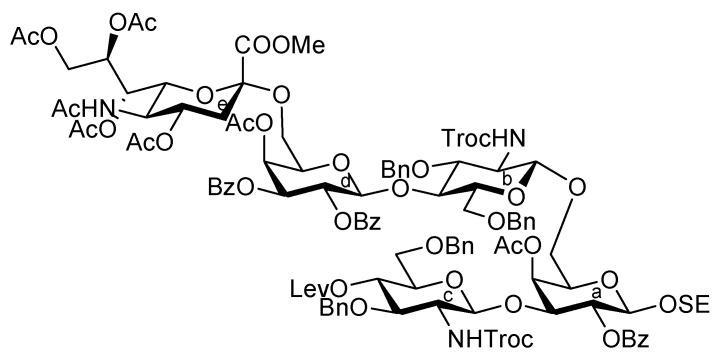



*2-(Trimethylsilyl)ethyl (methyl 5-acetamido-4,7,8,9-tetra-O-acetyl-3,5-dideoxy-D-glycero-α-D-galacto-2-nonulopyranosylonate)-(2→6)-4-O-acetyl-2,3-di-O-benzoyl-β-D-galactopyranosyl-(1→4)-3,6-di-O-benzyl-2-deoxy-2-(2,2,2-trichloroethoxycarbamoyl)-β-D-glucopyranosyl-(1→6)-[3,6-di-O-benzyl-2-deoxy-4-O-levulinoyl-2-(2,2,2-trichloroethoxycarbamoyl)-α-D-glucopyranosyl-(1→3)]-4-O-acetyl-2-O-benzoyl-β-D-galactopyranoside* (**29**). To a mixture of **12** (80 mg, 76.5 μmol) and **28** (70 mg, 45.0 μmol) in CH_2_Cl_2_ (0.9 mL) was added 3 Å molecular sieves (250 mg) at r.t. After stirring for 1 h, the mixture was cooled to 0 °C. TMSOTf (1.4 μL, 7.65 μmol) was then added to the mixture at 0 °C. After stirring for 25 h at r.t., TMSOTf (1.4 μL, 7.65 μmol) was added to the mixture. After the stirring was continued for 3 h at r.t. as the reaction was monitored by TLC (30:1 CHCl_3_–MeOH), the reaction was quenched by the addition of triethylamine. The mixture was diluted with CHCl_3_ and filtered through Celite. The filtrate was then washed with satd aq NaHCO_3_ and brine. The organic layer was subsequently dried over Na_2_SO_4_, and concentrated. The resulting residue was purified by silica gel column chromatography (90:1 CHCl_3_–MeOH) followed by gel filtration column chromatography (LH-20) using MeOH as eluent, giving **29** (81 mg, 74%). [α]_D_ −2.9° (c 1.4, CHCl_3_); ^1^H-NMR (600 MHz, CDCl_3_) δ 8.05–7.14 (m, 35 H, 7 Ph), 5.61 (d, 1 H, *J*_3,4_ = 3.3 Hz, H-4d), 5.59 (near t, 1 H, *J*_1,2_ = 8.3 Hz, H-2d), 5.45 (d, 1 H, *J*_3,4_ = 3.4 Hz, H-4a), 5.40–5.36 (m, 1 H, H-8e), 5.36 (t, 1 H, *J*_1,2_ = 8.2 Hz, H-2a), 5.34 (m, 1 H, H-7e), 5.30 (dd, 1 H, *J*_2,3_ = 10.2 Hz, H-3d), 5.23 (2 br d, 2 H, NHe, NHb), 5.02 (d, 1 H, *J*_2,NH_ = 6.9 Hz, NHc), 5.12 (d, 1 H, *J*_gem_ = 11.0 Hz, OCH_2_), 4.94 (d, 1 H, H-1d), 4.94–4.86 (m, 2 H, H-1c, H-4c), 4.84 (m, 1 H, *J*_3eq,4_ = 4.2 Hz, H-4e), 4.73–4.68 (m, 3 H, 3 OCH_2_), 4.58–4.30 (m, 10 H, H-1a, H-1b, H-9e, 7 OCH_2_), 4.14–3.71 (m, 15 H, H-3a, H-6'a, H-3b, H-4b, H-3c, H-4c, H-5d, H-6d, H-6'd, H-5e, H-6e, OCH_2_, OCH_3_), 3.58–3.37 (m, 10 H, H-6a, H-2b, H-5b, H-6b, H-6'b, H-5c, H-6c, H-6'c, 2 OCH_2_), 3.28–3.26 (m, 1 H, H-5a), 3.08 (br dd, 1 H, H-2c), 2.60–2.51 (m, 3 H, H-3e*eq*, CH_2_CH_2_C(=O)O), 2.38–2.24 (m, 2 H, CH_2_CH_2_C(=O)O), 2.16–1.91 (m, 25 H, 8 C(=O)CH_3_, H-3e*ax*), 0.85–0.69 (m, 2 H, CH_2_CH_2_SiMe_3_), −0.14 (s, 9 H, CH_2_CH_2_SiMe_3_); ^13^C-NMR (100 MHz, CDCl_3_) δ 206.0, 171.4, 170.9, 170.7, 170.3, 170.1, 169.8, 169.6, 167.8, 165.3, 165.2, 164.8, 153.9, 153.3, 138.6, 138.1, 138.0, 137.7, 133.3, 133.1, 129.8, 129.6, 129.5, 129.1, 128.5, 128.4, 128.4, 128.3, 128.3, 128.2, 127.9, 127.8, 127.7, 127.6, 127.6, 127.4, 100.6, 100.1, 99.8, 99.0, 95.4, 77.4, 76.8, 76.1, 74.4, 74.3, 73.9, 73.7, 73.3, 73.3, 73.1, 72.8, 71.7, 71.5, 71.4, 70.2, 70.1, 69.6, 68.7, 68.4, 68.1, 67.3, 62.4, 57.9, 57.0, 52.9, 49.4, 37.7, 37.6, 29.6, 27.8, 23.1, 21.0, 20.8, 20.5, 17.8, −1.5. *m/z* (MALDI): found [M+Na]^+^ 2463.09, C_113_H_131_Cl_6_N_3_O_42_Si calcd for [M+Na]^+^ 2462.60.



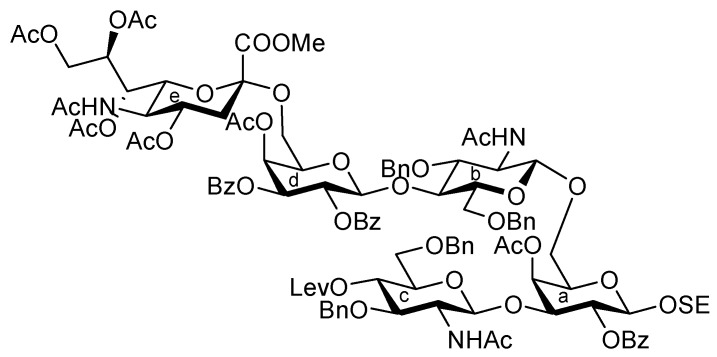



*2-(Trimethylsilyl)ethyl (methyl 5-acetamido-4,7,8,9-tetra-O-acetyl-3,5-dideoxy-D-glycero-α-D-galacto-2-nonulopyranosylonate)-(2→6)-4-O-acetyl-2,3-di-O-benzoyl-β-D-galactopyranosyl-(1→4)-2-acetamido-3,6-di-O-benzyl-2-deoxy-β-D-glucopyranosyl-(1→6)-[2-acetamido-3,6-di-O-benzyl-2-deoxy-4-O-levulinoyl-β-D-glucopyranosyl-(1→3)]-4-O-acetyl-2-O-benzoyl-β-D-galactopyranoside* (**31**). To a solution of **29** (220 mg, 89.9 μmol) in CH_2_Cl_2_/AcOH (2:3, 3.5 mL) was added Zn/Cu couple (2.20 g) at r.t. The reaction mixture was heated to 50 °C and was stirred for 2.5 h at the same temperature as the reaction was monitored by TLC (15:1 CHCl_3_–MeOH). The precipitate was filtered through Celite and the filtrate was co-evaporated with toluene. The obtained residue was exposed to high vacuum for 6 h. The crude residue was dissolved in pyridine (3.6 mL) and acetic anhydride (68 μL, 0.719 mmol), DMAP (2.5 mg, 47.6 μmol) were then added to the mixture at 0 °C. After stirring for 12 h at r.t as the reaction was monitored by TLC (15:1 CHCl_3_–MeOH), the reaction mixture was evaporated. The obtained residue was diluted with CHCl_3_ and washed with 2 M HCl, satd aq NaHCO_3_, and brine, dried over Na_2_SO_4_, and concentrated. The resulting residue was purified by silica gel column chromatography (40:1 CHCl_3_–MeOH) to give **31** (120 mg, 61%). [α]_D_ −1.9° (c 0.5, CHCl_3_); ^1^H-NMR (600 MHz, CDCl_3_) δ 8.02–7.13 (m, 35 H, 7 Ph), 5.93 (d, 1 H, *J*_2,NH_ = 8.3 Hz, NHb), 5.63 (d, 1 H, *J*_3,4_ = 3.4 Hz, H-4d), 5.56 (near t, 1 H, *J*_1,2_ = 7.5 Hz, *J*_2,3_ = 8.2 Hz, H-2d), 5.41–5.31 (m, 5 H, H-2a, H-4a, H-3d, H-7e, H-8e), 5.26 (d, 1 H, *J*_2,NH_ = 7.6 Hz, NHc), 5.13 (d, 1 H, *J*_5,NH_ = 7.5 Hz, NHe), 4.95 (d, 1 H, *J*_1,2_ = 8.2 Hz, H-1c), 4.90–4.83 (m, 4 H, H-4c, H-1d, H-4e, OCH_2_), 4.70 (d, 1 H, *J*_gem_ = 11.7 Hz, OCH_2_), 4.58 (d, 1 H, *J*_1,2_ = 6.9 Hz, H-1c), 4.44 (d, 1 H, *J*_1,2_ = 8.3 Hz, H-1a), 4.54–4.31 (m, 7 H, H-9e, 3 OCH_2_), 4.13 (t, 1 H, *J*_2,3_ = 9.6 Hz, H-3c), 4.10–4.02 (m, 4 H, H-6a, H-4b, H-5e, H-9e), 3.97–3.75 (m, 9 H, H-3a, H-6'a, H-3b, H-6d, H-6e, OCH_2_CH_2_SiMe_3_, OCH_3_), 3.67–3.35 (m, 11 H, H-5a, H-2b, H-5b, H-6b, H-6'b, H-5c, H-6c, H-6'c, H-5d, H-6'd, OCH_2_CH_2_SiMe_3_), 3.07 (dd, 1 H, H-2c), 2.54–2.51 (m, 3 H, H-3e*eq*, CH_2_CH_2_C(=O)O), 2.35–2.27 (m, 2 H, CH_2_CH_2_C(=O)O), 2.15–1.89 (m, 28 H, 9 C(=O)CH_3_, H-3e*ax*), 0.88–0.69 (m, 2 H, OCH_2_CH_2_SiMe_3_), −0.16 (s, 9 H, OCH_2_CH_2_SiMe_3_); ^13^C (125 MHz, CDCl_3_) δ 206.1, 171.5, 170.9, 170.7, 170.5, 170.3, 170.1, 169.9, 169.7, 167.8, 165.3, 165.1, 138.9, 138.2, 138.1, 133.4, 133.3, 133.2, 129.8, 129.7, 129.6, 129.1, 128.6, 128.5, 128.5, 128.4, 128.3, 127.9, 127.9, 127.8, 127.7, 127.6, 127.5, 127.4, 100.6, 100.2, 99.9, 99.7, 99.0, 78.0, 77.4, 74.6, 73.6, 73.4, 73.3, 73.2, 73.1, 72.7, 71.8, 71.8, 71.6, 71.5, 70.2, 70.1, 69.7, 68.8, 68.8, 68.1, 67.6, 67.4, 67.2, 62.7, 62.5, 57.6, 52.9, 49.5, 37.8, 37.7, 29.7, 27.9, 23.4, 23.2, 22.7, 21.0, 20.8, 20.7, 20.6, 17.8, −1.5. *m/z* (MALDI): found [M+Na]^+^ 2198.83, C_111_H_133_N_3_O_40_Si calcd for [M+Na]^+^ 2198.81.



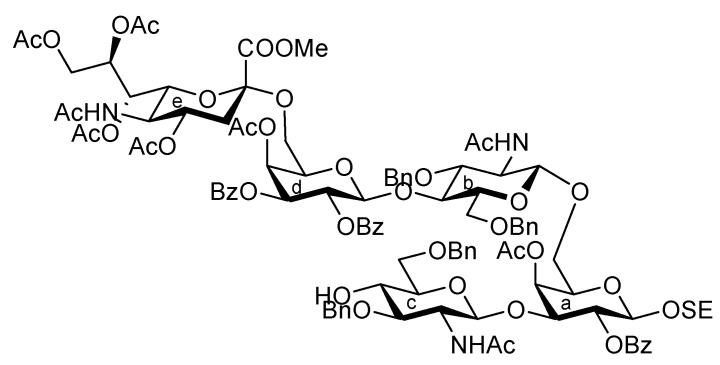



*2-(Trimethylsilyl)ethyl (methyl 5-acetamido-4,7,8,9-tetra-O-acetyl-3,5-dideoxy-D-glycero-α-D-galacto-2-nonulopyranosylonate)-(2→6)-4-O-acetyl-2,3-di-O-benzoyl-β-D-galactopyranosyl-(1→4)-2-acetamido-3,6-di-O-benzyl-2-deoxy-β-D-glucopyranosyl-(1→6)-[2-acetamido-3,6-di-O-benzyl-2-deoxy-β-D-glucopyranosyl-(1→3)]-4-O-acetyl-2-O-benzoyl-β-D-galactopyranoside* (**32**). To a solution of **31** (119 mg, 54.7 μmol) in THF (2.0 mL) was added hydrazine monoacetate (4.9 mg, 54.7 μmol) at 0 °C. After stirring for 1 h at r.t. as the reaction was monitored by TLC (30:1 CHCl_3_–MeOH), THF was evaporated. The residue was diluted with CHCl_3_ and washed with 2 M HCl, satd aq NaHCO_3_, and brine, dried over Na_2_SO_4_, and concentrated. The obtained residue was purified by silica gel column chromatography (40:1 CHCl_3_–MeOH) to give **32** (104 mg, 92%). [α]_D_ −5.2° (c 1.9, CHCl_3_); ^1^H-NMR (500 MHz, CDCl_3_) δ 8.02–7.20 (m, 35 H, 7 Ph), 6.01 (d, 1 H, *J*_NH,2_ = 8.6 Hz, NHb), 5.64 (d, 1 H, *J*_3,4_ = 3.5 Hz, H-4d), 5.58 (near t, 1 H, *J*_1,2_ = 8.0 Hz, *J*_2,3_ = 10.3 Hz, H-2d), 5.43–5.32 (m, 6 H, H-2a, H-4a, H-3d, H-7e, H-8e, NHc), 5.21 (d, 1 H, *J*_NH,5_ = 7.4 Hz, NHe), 4.89–4.81 (m, 3 H, H-1d, H-4e, OCH_2_), 4.82 (d, 1 H, *J*_1,2_ = 9.7 Hz, H-1c), 4.72–4.65 (2 d, 2 H, *J*_gem_ = 11.5 Hz, 2 OCH_2_), 4.60–4.45 (m, 5 H, H-1b, 4 OCH_2_), 4.44 (d, 1 H, *J*_1,2_ = 8.0 Hz, H-1a), 4.37–4.32 (m, 2 H, OCH_2_, H-9e), 4.12–4.04 (m, 4 H, H-6a, H-6d, H-6e, H-9'e), 3.98–3.62 (m, 14 H, H-3a, H-5a, H-6'a, H-2b, H-3b, H-6b, H-6'b, H-3c, H-5d, H-6'd, OCH_3_, OCH_2_CH_2_SiMe_3_), 3.54 (t, 1 H, *J*_3,4_ = *J*_4,5_ = 12.0 Hz, H-4c), 3.49–3.36 (m, 6 H, H-5c, H-6c, H-6'c, H-4b, H-5b, OCH_2_CH_2_SiMe_3_), 3.16 (dd, 1 H, H-2c), 2.96 (s, 1 H, OH), 2.53 (dd, 1 H, *J*_gem_ = 12.4 Hz, *J*_3eq,4_ = 4.6 Hz, H-3e*eq*), 2.18–1.87 (m, 25 H, 8 C(=O)CH_3_, H-3e*ax*), 0.88–0.69 (m, 2 H, OCH_2_CH_2_SiMe_3_), −0.16 (s, 9 H, OCH_2_CH_2_SiMe_3_); ^13^C-NMR (125 MHz, CDCl_3_) δ 170.8, 170.6, 170.4, 170.2, 170.1, 169.9, 169.6, 169.5, 167.7, 165.6, 165.2, 165.0, 138.7, 138.4, 138.0, 137.7, 133.4, 133.2, 133.1, 129.7, 129.7, 129.6, 129.5, 129.0, 128.4, 128.4, 128.3, 128.3, 128.2, 127.8, 127.8, 127.7, 127.6, 127.5, 127.3, 100.4, 100.2, 100.1, 99.6, 98.9, 79.8, 77.9, 75.2, 74.5, 73.8, 73.5, 73.5, 73.2, 73.1, 73.0, 72.8, 72.6, 71.7, 71.6, 71.4, 70.7, 70.1, 70.0, 68.7, 68.1, 67.5, 67.3, 67.2, 62.5, 62.4, 56.4, 54.0, 52.8, 49.2, 37.7, 29.6, 23.3, 23.1, 22.8, 20.9, 20.7, 20.6, 20.5, 17.7, −1.6. *m/z* (MALDI): found [M+Na]^+^ 2100.96, C_106_H_127_N_3_O_38_Si calcd for [M+Na]^+^ 2100.78.



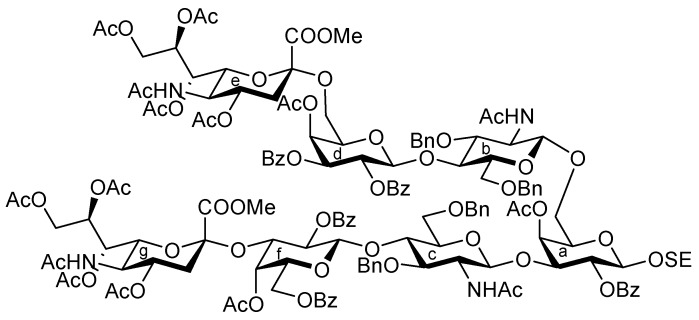



*2-(Trimethylsilyl)ethyl (methyl 5-acetamido-4,7,8,9-tetra-O-acetyl-3,5-dideoxy-D-glycero-α-D-galacto-2-nonulopyranosylonate)-(2→3)-4-O-acetyl-2,6-di-O-benzoyl-β-D-galactopyranosyl-(1→4)-2-acetamido-3,6-di-O-benzyl-2-deoxy-β-D-glucopyranosyl-(1→3)-[(methyl 5-acetamido-4,7,8,9-tetra-O-acetyl-3,5-dideoxy-D-glycero-α-D-galacto-2-nonulopyranosylonate)-(2→6)-4-O-acetyl-2,3-di-O-benzoyl-β-D-galactopyranosyl-(1→4)-2-acetamido-3,6-di-O-benzyl-2-deoxy-β-D-glucopyranosyl-(1→6)]-4-O-acetyl-2-O-benzoyl-β-D-galactopyranoside* (**33**). To a mixture of **4** (103 mg, 98.7 μmol) and **32** (93 mg, 44.5 μmol) in CH_2_Cl_2_ (1.8 mL) was added 3 Å molecular sieves (300 mg) at r.t. After stirring for 1 h, the mixture was cooled to 0 °C. TMSOTf (1.8 μL, 9.94 μmol) was then added to the mixture at 0 °C. The reaction was stirred for 2 h at r.t. as the reaction was monitored by TLC (15:1:1 CHCl_3_–MeOH–EtOAc). Another portion of TMSOTf (1.8 μL, 9.94 μmol) was added to the mixture at 0 °C. After the stirring was continued for 4 h at r.t, the reaction was quenched by the addition of triethylamine. The reaction mixture was diluted with CHCl_3_ and filtered through Celite. The filtrate was then washed with satd aq NaHCO_3_ and brine. The organic layer was subsequently dried over Na_2_SO_4_, and concentrated. The resulting residue was purified by silica gel column chromatography (40:1 CHCl_3_–MeOH) to give **33** (82 mg, 62%). [α]_D_ −5.1° (c 0.8, CHCl_3_); ^1^H-NMR (600 MHz, CDCl_3_) δ 8.22–7.12 (m, 45 H, 9 Ph), 5.92 (d, 1 H, NHb), 5.65 (m, 1 H, H-4g), 5.63 (d, 1 H, *J*_3,4_ = 3.4 Hz, H-4d), 5.55 (near t, 1 H, *J*_1,2_ = 8.2 Hz, *J*_2,3_ = 10.2 Hz, H-2d), 5.40 (m, 1 H, H-8e), 5.37 (dd, 1 H, H-3d), 5.33–5.13 (m, 7 H, H-2a, H-4a, H-7e, H-2f, H-7g, NHc, NHe), 5.06 (d, 1 H, *J*_3,4_ = 3.4 Hz, H-4f), 5.01 (d, 1 H, *J*_1,2_ = 7.5 Hz, H-1f), 5.00 (d, 1 H, *J*_5,NH_ = 7.5 Hz, NHg), 4.87–4.82 (m, 5 H, H-1d, H-4e, H-4g, 2 OCH_2_), 4.75 (dd, 1 H, *J*_2,3_ = 10.2 Hz, H-3f), 4.71 (d, 1 H, *J*_gem_ = 11.6 Hz, OCH_2_), 4.68 (d, 1 H, *J*_1,2_ = 7.5 Hz, H-1c), 4.68–4.47 (m, 4 H, H-1b, 3 OCH_2_), 4.42 (d, 1 H, *J*_1,2_ = 10.7 Hz, H-1a), 4.28 (m, 4 H, H-9e, H-9g, 2 OCH_2_), 4.11–4.00 (m, 7 H, H-4b, H-4c, H-6c, H-6'c, H-5e, H-6e, H-9'e), 3.97–3.55 (m, 23 H, H-3a, H-5a, H-6a, H-6'a, H-2b, H-3b, H-6b, H-6'b, H-3c, H-6d, H-6'd, H-6f, H-6'f, H-5g, H-6g, H-9'g, 2 OCH_3_, OCH_2_CH_2_SiMe_3_), 3.49–3.40 (m, 3 H, H-5b, H-5c, OCH_2_CH_2_SiMe_3_), 3.31–3.28 (m, 2 H, H-5c, H-5f), 3.14 (br dd, 1 H, H-2c), 2.53 (dd, 1 H, *J*_gem_ = 11.3 Hz, *J*_3eq,4_ = 4.8 Hz, H-3g*eq*), 2.49 (m, 1 H, *J*_gem_ = 11.7 Hz, *J*_3eq,4_ = 4.8 Hz, H-3e*eq*), 2.15–1.74 (m, 43 H, 14 Ac, H-3e*ax*), 1.70 (t, 1 H, H-3g*ax*), 1.51 (s, 3 H, Ac), 0.88–0.68 (m, 2 H, OCH_2_CH_2_SiMe_3_), −0.17 (s, 9 H, OCH_2_CH_2_SiMe_3_); ^13^C-NMR (150 MHz, CDCl_3_) δ 170.9, 170.7, 170.7, 170.3, 170.2, 170.1, 170.1, 170.0, 169.8, 169.7, 169.6, 168.0, 167.8, 165.7, 165.5, 165.3, 165.2, 164.9, 138.8, 138.8, 138.7, 138.1, 133.4, 133.3, 133.2, 133.1, 133.0, 130.3, 129.9, 129.8, 129.7, 129.7, 129.6, 129.5, 129.0, 128.6, 128.5, 128.4, 128.2, 128.1, 128.0, 127.9, 129.6, 129.5, 129.0, 128.6, 128.5, 128.4, 128.2, 128.1, 128.0, 127.9, 127.8, 127.5, 127.4, 127.2, 127.2, 127.0, 100.5, 100.3, 99.6, 99.5, 99.0, 96.8, 78.1, 77.6, 77.5, 75.2, 75.0, 74.6, 74.5, 73.6, 73.3, 73.0, 72.7, 72.6, 71.8, 71.7, 71.4, 71.3, 70.4, 70.2, 70.1, 69.4, 68.7, 68.1, 68.0, 67.5, 67.3, 67.2, 67.1, 66.6, 62.4, 61.3, 53.0, 52.9, 49.4, 48.8, 37.8, 37.3, 29.7, 23.3, 23.2, 23.1, 22.6, 21.3, 21.0, 20.8, 20.8, 20.7, 20.6, 20.6, 20.3, 17.7, −1.5. *m/z* (MALDI): found [M+Na]^+^ 2986.28, C_148_H_174_N_4_O_58_Si calcd for [M+Na]^+^ 2986.05.



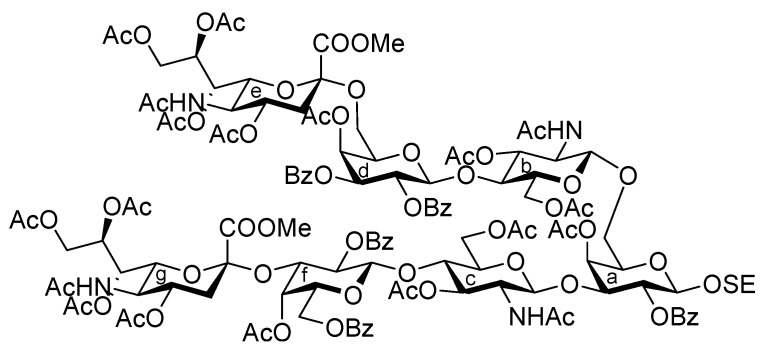



*2-(Trimethylsilyl)ethyl (methyl 5-acetamido-4,7,8,9-tetra-O-acetyl-3,5-dideoxy-D-glycero-α-D-galacto-2-nonulopyranosylonate)-(2→3)-4-O-acetyl-2,6-di-O-benzoyl-β-D-galactopyranosyl-(1→4)-2-acetamido-3,6-di-O-acetyl-2-deoxy-β-D-glucopyranosyl-(1→3)-[(methyl 5-acetamido-4,7,8,9-tetra-O-acetyl-3,5-dideoxy-D-glycero-α-D-galacto-2-nonulopyranosylonate)-(2→6)-4-O-acetyl-2,3-di-O-benzoyl-β-D-galactopyranosyl-(1→4)-2-acetamido-3,6-di-O-acetyl-2-deoxy-β-D-glucopyranosyl-(1→6)]-4-O-acetyl-2-O-benzoyl-β-D-galactopyranoside* (**35**). To a solution of **33** (42.0 mg, 14.2 μmol) in 1,4-dioxane (0.6 mL) was added Pd(OH)_2_/C (210 mg). After stirring for 4 h at r.t. under a hydrogen atmosphere as the reaction was monitored by TLC (10:1 CHCl_3_–MeOH), the mixture was filtered through Celite. The filtrate was concentrated and the obtained crude residue was roughly purified by silica gel column chromatography (10:1 CHCl_3_–MeOH). The obtained product was exposed to high vacuum for 24 h. The residue was then dissolved in pyridine (1.4 mL). Acetic anhydride (11 μL, 0.114 mmol) and DMAP (1.0 mg, 8.18 μmol) were added to the mixture at 0 °C. After stirring for 72 h at r.t. as the reaction was monitored by TLC (10:1 CHCl_3_–MeOH), the reaction was quenched by the addition of MeOH at 0 °C. The mixture was co-evaporated with toluene and the residue was then diluted with CHCl_3_, and washed with 2 M HCl, H_2_O, satd aq NaHCO_3_ and brine. The organic layer was subsequently dried over Na_2_SO_4_, and concentrated. The resulting residue was purified by silica gel column chromatography (40:1 CHCl_3_–MeOH) to give **35** (35 mg, 89%). [α]_D_ +5.1° (c 0.7, CHCl_3_); ^1^H-NMR (500 MHz, CDCl_3_) δ 8.17–7.27 (m, 25 H, 5 Ph), 5.92 (d, 1 H, *J*_NH,2_ = 9.2 Hz, NHb), 5.68 (d, 1 H, *J*_3,4_ = 3.5 Hz, H-4d), 5.77 (m, 1 H, H-8g), 5.55 (near t, 2 H, *J*_1,2_ = 8.0 Hz, *J*_2,3_ = 10.3 Hz, H-2d), 5.44 (m, 1 H, H-3d), 5.38 (m, 1 H, H-8e), 5.32–5.16 (m, 7 H, H-2a, H-3a, H-3b, H-7e, H-2f, H-7g, NHe), 5.09 (d, 1 H, *J*_3,4_ = 2.8 Hz, H-4f), 4.96 (d, 1 H, *J*_NH,5_ = 9.8 Hz, NHg), 4.91–4.80 (m, 6 H, NHc, H-3c, H-1d, H-4e, H-1f, H-4g), 4.74–4.71 (dd, 1 H, *J*_2,3_ = 9.7 Hz, H-3f), 4.47 (d, 1 H, *J*_1,2_ = 8.0 Hz, H-1b), 4.43 (d, 1 H, *J*_1,2_ = 8.0 Hz, H-1a), 4.41–4.28 (m, 6 H, H-6a, H-1c, H-6d, H-9e, H-6f, H-9g), 4.17–3.93 (m, 10 H, H-6'a, H-6b, H-6'b, H-6'd, H-5e, H-6e, H-9'e, H-6'f, H-9'g, OCH_2_CH_2_SiMe_3_), 3.88–3.68 (m, 15 H, H-3a, H-2b, H-4b, H-5b, H-2c, H-4c, H-6c, H-6'c, H-5g, 2 OCH_3_), 3.55–3.53 (m, 2 H, H-5d, H-6g), 3.48–3.42 (m, 3 H, H-5a, H-5f, OCH_2_CH_2_SiMe_3_), 3.27–3.25 (m, 1 H, *J*_5,4_ = 9.7 Hz, H-5c), 2.55 (dd, 1 H, *J*_gem_ = 12.6 Hz, *J*_3eq,4_ = 4.6 Hz, H-3g*eq*), 2.51 (dd, 1 H, *J*_gem_ = 12.6 Hz, *J*_3eq,4_ = 4.6 Hz, H-3e*eq*), 2.20–1.78 (m, 52 H, 17 Ac, H-3e*ax*), 1.62 (t, 1 H, H-3g*ax*), 1.54 (s, 6 H, 2 Ac), 0.88–0.72 (m, 2 H, OCH_2_CH_2_SiMe_3_), −0.15 (s, 9 H, OCH_2_CH_2_SiMe_3_); ^13^C-NMR (150 MHz, CDCl_3_) δ 170.9, 170.7, 170.7, 170.5, 170.4, 170.4, 170.2, 170.1, 170.1, 170.0, 169.8, 169.8, 169.7, 168.0, 167.7, 165.6, 165.3, 165.0, 164.9, 164.8, 133.5, 133.3, 133.2, 130.3, 129.8, 129.7, 129.5, 129.0, 128.9, 128.7, 128.5, 128.5, 128.4, 128.4, 101.2, 100.7, 100.6, 100.5, 100.4, 99.2, 96.8, 75.6, 74.6, 72.9, 72.7, 72.5, 72.4, 71.9, 71.7, 71.1, 70.9, 70.0, 69.8, 69.6, 69.4, 68.6, 67.8, 67.4, 67.1, 66.2, 62.9, 62.4, 62.2, 62.0, 61.0, 60.9, 53.9, 53.0, 49.4, 48.8, 37.8, 37.2, 23.2, 23.1, 22.7, 21.3, 21.0, 20.9, 20.8, 20.7, 20.6, 20.5, 20.4, 17.8, −1.5. *m/z* (MALDI): found [M+Na]^+^ 2793.65, C_128_H_158_N_4_O_62_Si calcd for [M+Na]^+^ 2793.90.



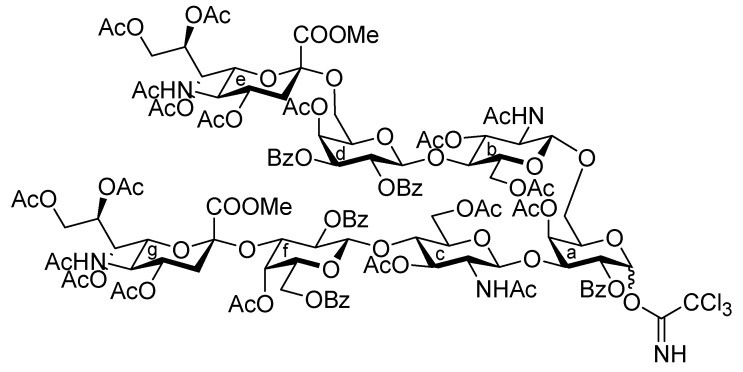



*(Methyl 5-acetamido-4,7,8,9-tetra-O-acetyl-3,5-dideoxy-D-glycero-α-D-galacto-2-nonulopyranosylonate)-(2→3)-4-O-acetyl-2,6-di-O-benzoyl-β-D-galactopyranosyl-(1→4)-2-acetamido-3,6-di-O-acetyl-2-deoxy-β-D-glucopyranosyl-(1→3)-[(methyl 5-acetamido-4,7,8,9-tetra-O-acetyl-3,5-dideoxy-D-glycero-α-D-galacto-2-nonulopyranosylonate)-(2→6)-4-O-acetyl-2,3-di-O-benzoyl-β-D-galactopyranosyl-(1→4)-2-acetamido-3,6-di-O-acetyl-2-deoxy-β-D-glucopyranosyl-(1→6)]-4-O-acetyl-2-O-benzoyl-D-galactopyranosyl trichloroacetimidate* (**37**). To a solution of **35** (28 mg, 10.2 μmol) in CH_2_Cl_2_ (0.75 mL) was added TFA (0.25 mL) at 0 °C. After stirring for 2 h at 0 °C as the reaction was monitored by TLC (15:1:1 CHCl_3_–MeOH–EtOAc), the reaction mixture was co-evaporated with toluene and then roughly purified by silica gel column chromatography (20:1 CHCl_3_–MeOH). The obtained product was exposed to high vacuum for 24 h and then dissolved in CH_2_Cl_2_ (1.0 mL). CCl_3_CN (10.2 μL, 0.102 mmol) and DBU (1.8 μL, 12.2 μmol) were added to the mixture at 0 °C. After stirring for 45 min at 0 °C as the reaction was monitored by TLC (10:1 CHCl_3_–MeOH), the reaction mixture was evaporated. The obtained crude residue was purified by silica gel column chromatography (30:1 CHCl_3_–MeOH) to give **37** (27 mg, 95%, α:β = 3:1). **37α**: ^1^H-NMR (600 MHz, CDCl_3_) δ 8.60 (s, 1 H, C(=NH)), 8.19–7.27 (m, 25 H, 5 Ph), 6.48 (d, 1 H, *J*_1,2_ = 4.1 Hz, H-1a), 5.81 (d, 1 H, *J*_NH,2_ = 9.0 Hz, NHc), 5.68 (d, 1 H, *J*_3,4_ = 3.5 Hz, H-4d), 5.60 (m, 1 H, H-8g), 5.55 (near t, 1 H, *J*_1,2_ = 8.3 Hz, *J*_2,3_ = 10.3 Hz, H-2d), 5.48 (dd, 1 H, *J*_2,3_ = 10.3 Hz, H-2a), 5.43–5.42 (m, 2 H, H-3a, H-4a), 5.38 (m, 1 H, H-8e), 5.32 (dd, 1 H, H-7e), 5.24 (m, 2 H, H-3a, H-7g), 5.20 (near t, 1 H, *J*_1,2_ = 8.3 Hz, *J*_2,3_ = 9.6 Hz, H-2f), 5.16 (t, 1 H, *J*_2,3_ = *J*_3,4_ = 9.0 Hz, H-3b), 5.13 (d, 1 H, *J*_5,NH_ = 9.6 Hz, NHe), 4.92 (d, 1 H, *J*_3,4_ = 2.3 Hz, H-4f), 4.90–4.80 (m, 8 H, H-3c, H-1d, H-4e, H-1f, H-3d, H-4g, NHc, NHg), 4.73 (dd, 1 H, H-3f), 4.55 (d, 1 H, *J*_1,2_ = 7.6 Hz, H-1b), 4.45 (d, 1 H, *J*_1,2_ = 7.4 Hz, H-1c), 4.41–4.01 (m, 13 H, H-3a, H-6a, H-6'a, H-5e, H-6e, H-9e, H-9'e, H-6d, H-6'd, H-6f, H-6'f, H-9g, H-9'g), 3.88–3.70 (m, 15 H, H-2b, H-4b, H-6b, H-6'b, H-2c, H-4c, H-6c, H-6'c, H-5e, 2 OCH_3_), 3.55–3.36 (m, 5 H, H-5a, H-5b, H-5c, H-5d, H-5f), 2.55 (dd, 1 H, *J*_gem_ = 13.1 Hz, *J*_3eq,4_ = 4.8 Hz, H-3e*eq* ), 2.51 (dd, 1 H, *J*_gem_ = 11.6 Hz, *J*_3eq,4_ = 4.8 Hz, H-3g*eq*), 2.20–1.78 (m, 52 H, 17 Ac, H-3e*ax*), 1.63–1.39 (m, 7 H, 2 Ac, H-3g*ax*).



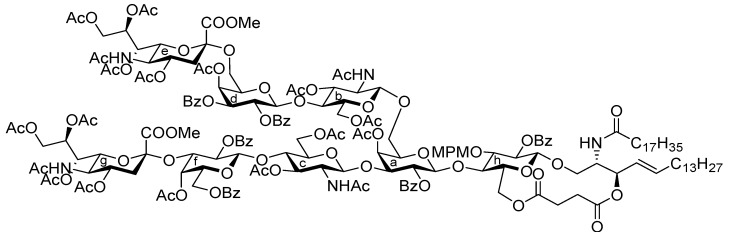



*(2S,3R,4E)-1-O-({(Methyl 5-acetamido-4,7,8,9-tetra-O-acetyl-3,5-dideoxy-D-glycero-α-D-galacto-2-nonulopyranosylonate)-(2→3)-4-O-acetyl-2,6-di-O-benzoyl-β-D-galactopyranosyl-(1→4)-2-acetamido-3,6-di-O-acetyl-2-deoxy-β-D-glucopyranosyl-(1→3)-[(methyl 5-acetamido-4,7,8,9-tetra-O-acetyl-3,5-dideoxy-D-glycero-α-D-galacto-2-nonulopyranosylonate)-(2→6)-4-O-acetyl-2,3-di-O-benzoyl-β-D-galactopyranosyl-(1→4)-2-acetamido-3,6-di-O-acetyl-2-deoxy-β-D-glucopyranosyl-(1→6)]-4-O-acetyl-2-O-benzoyl-β-D-galactopyranosyl}-(1→4)-2-O-benzoyl-3-O-p-methoxybenzyl-β-D-glucopyranosyl)-2-octadecanamido-3,6´-succinyl-octadec-4-ene-1,3-diol* (**39**). To a mixture of **37** (38 mg, 13.4 μmol) and **38** (14 mg, 13.4 μmol) in CH_2_Cl_2_ (1.3 mL) was added 5 Å molecular sieves (100 mg) at r.t. After stirring for 1 h, the mixture was cooled to 0 °C. TMSOTf (0.3 μL, 1.34 μmol) was then added to the mixture at 0 °C. After stirring for 1 h at r.t. as the reaction was monitored by TLC (15:1:5 CHCl_3_–MeOH–EtOAc), the reaction was quenched by the addition of triethylamine. The reaction mixture was diluted with CHCl_3_ and filtered through Celite. The filtrate was then washed with satd aq NaHCO_3_ and brine. The organic layer was subsequently dried over Na_2_SO_4_, and concentrated. The residue was purified by silica gel column chromatography (40:1:5 CHCl_3_–MeOH–EtOAc) to give **39** (24 mg, 49%). [α]_D_ −10.3° (c 2.0, CHCl_3_); ^1^H-NMR (500 MHz, CD_3_CN) δ 8.13–7.39 (m, 30 H, 6 Ph), 7.04 (d, 2 H, Ar), 6.66 (d, 2 H, Ar), 6.14 (d, 1 H, *J*_5,NH_ = 9.7 Hz, NHe), 6.09 (d, 1 H, *J*_2,NH_ = 9.2 Hz, NH*^Cer^*), 6.00 (d, 1 H, *J*_5,NH_ = 9.7 Hz, NHg), 5.95 (d, 1 H, *J*_2,NH_ = 9.1 Hz, NHb), 5.79 (d, 1 H, *J*_2,NH_ = 9.8 Hz, NHc), 5.63 (dt, 1 H, *J*_5,6_ = 6.9 Hz, *J* = 14.9 Hz, *J* = 6.9 Hz, H-5*^Cer^*), 5.54 (d, 1 H, *J*_3,4_ = 3.5 Hz, H-4d), 5.47 (m, 3 H, H-3d, H-7e, H-8e), 5.37 (near t, 1 H, *J*_1,2_ = 7.5 Hz, *J*_2,3_ = 10.3 Hz, H-2d), 5.36 (m, 1 H, H-8g), 5.27 (d, 1 H, *J*_3,4_ = 3.5 Hz, H-4a), 5.25–5.17 (m, 4 H, H-2a, H-4f, H-7g, H-4*^Cer^*), 5.11 (br t, 1 H, *J*_3,4_ = *J*_2,3_ = 7.5 Hz, H-3*^Cer^*), 5.03 (near t, 1 H, *J*_1,2_ = 8.0 Hz, *J*_2,3_ = 10.3 Hz, H-2f), 4.94 (t, 1 H, *J*_1,2_ = *J*_2,3_ = 8.3 Hz, H-2h), 4.88 (t, 1 H, *J*_2,3_ = *J*_3,4_ = 9.3 Hz, H-3b), 4.76 (m, 8 H, H-1c, H-3c, H-1d, H-4e, H-1f, H-3f, H-4g, ArC*H_2_*), 4.68 (d, 1 H, *J*_1,2_ = 8.1 Hz, H-1a), 4.54 (d, 1 H, *J*_1,2_ = 8.5 Hz, H-1b), 4.47 (d, 1 H, H-1h), 4.46 (d, 1 H, *J*_gem_ = 10.9 Hz, ArC*H_2_*), 4.35 (dd, 1 H, *J*_8,9_ = 2.9 Hz, *J*_gem_ = 12.6 Hz, H-9e), 4.30–4.21 (m, 4 H, H-6b, H-6'b, H-6d, H-9g), 4.11–3.89 (m, 13 H, H-3a, H-6'a, H-6c, H-6'c, H-6'd, H-5e, H-6e, H-9'e, H-6f, H-9g, H-6h, H-6'h, H-2*^Cer^*), 3.78–3.53 (m, 21 H, H-5a, H-4b, H-2c, H-4c, H-5d, H-6'a, H-6'f, H-5g, H-6g, H-3h, H-4h, H-1*^Cer^*, 3 OCH_3_), 3.51 (m, 1 H, H-2b), 3.40–3.32 (m, 3 H, H-5b, H-5h, H-1'*^Cer^*), 3.18–3.12 (m, 2 H, H-5c, H-5f), 2.56 (dd, 1 H, *J*_3eq,4_ = 4.6 Hz, *J*_gem_ = 12.6 Hz, H-3e*eq*), 2.48–2.34 (m, 5 H, H-3g*eq*, 2 C(=O)CH_2_), 2.21–1.61 (m, 62 H, H-3e*ax*, H-6*^Cer^*, H-6'*^Cer^*, C(=O)CH_2_*^Cer^*, 19 Ac), 1.55–1.08 (m, 53 H, H-3g*ax*, 26 –CH_2_–), 0.87 (t, 6 H, 2 –CH_3_*^Cer^*); ^13^C-NMR (150 MHz, CDCl_3_) δ 172.6, 171.0, 170.9, 170.8, 170.7, 170.6, 170.5, 170.4, 170.3, 170.2, 170.1, 170.0, 169.7, 169.7, 169.5, 168.0, 167.7, 165.6, 165.2, 165.2, 165.1, 165.0, 164.5, 159.1, 138.6, 133.8, 133.3, 133.1, 130.7, 130.3, 129.8, 129.7, 129.6, 129.5, 129.5, 129.5, 129.0, 128.9, 128.8, 128.6, 128.6, 128.5, 128.4, 128.3, 124.8, 113.9, 101.6, 101.3, 100.8, 100.6, 100.3, 99.7, 99.3, 96.8, 91.8, 80.8, 79.7, 76.3, 75.9, 74.7, 74.3, 73.2, 72.9, 72.7, 72.6, 72.5, 71.9, 71.8, 71.7, 71.7, 71.0, 70.9, 70.1, 69.8, 69.7, 69.4, 68.6, 67.8, 67.4, 67.1, 66.3, 63.2, 63.0, 62.6, 62.1, 61.9, 61.1, 60.9, 55.1, 53.6, 53.1, 53.0, 49.9, 49.4, 48.8, 37.9, 37.2, 37.1, 36.6, 32.7, 32.2, 31.9, 30.0, 29.7, 29.6, 29.5, 29.5, 29.3, 29.3, 29.2, 28.8, 27.1, 25.5, 23.6, 23.2, 23.1, 22.7, 21.3, 21.0, 20.9, 20.8, 20.7, 20.7, 20.6, 20.5, 20.5, 19.7, 14.1. *m/z* (MALDI): found [M+Na]^+^ 3709.15, C_184_H_239_N_5_O_73_ calcd for [M+Na]^+^ 3709.50.



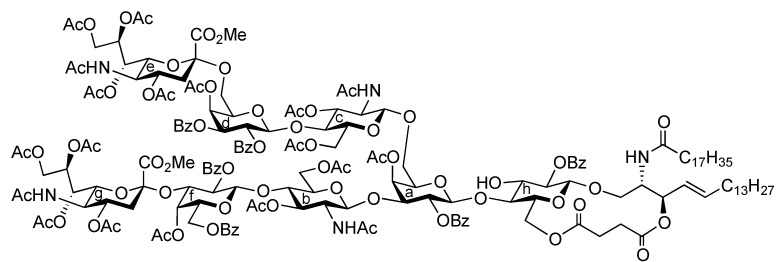



*(2S,3R,4E)-1-O-({(Methyl 5-acetamido-4,7,8,9-tetra-O-acetyl-3,5-dideoxy-D-glycero-α-D-galacto-2-nonulopyranosylonate)-(2→3)-4-O-acetyl-2,6-di-O-benzoyl-β-D-galactopyranosyl-(1→4)-2-acetamido-3,6-di-O-acetyl-2-deoxy-β-D-glucopyranosyl-(1→3)-[(methyl 5-acetamido-4,7,8,9-tetra-O-acetyl-3,5-dideoxy-D-glycero-α-D-galacto-2-nonulopyranosylonate)-(2→6)-4-O-acetyl-2,3-di-O-benzoyl-β-D-galactopyranosyl-(1→4)-2-acetamido-3,6-di-O-acetyl-2-deoxy-β-D-glucopyranosyl-(1→6)]-4-O-acetyl-2-O-benzoyl-β-D-galactopyranosyl}-(1→4)-2-O-benzoyl-β-D-glucopyranosyl)-2-octadecanamido-3,6'-succinyl-octadec-4-ene-1,3-diol* (**40**). To a solution of **39** (9.0 mg, 2.47 μmol) in CH_2_Cl_2_ (0.60 mL) was added TFA (0.30 mL) at 0 °C. After stirring for 30 min at 0 °C as the reaction was monitored by TLC (10:1 CHCl_3_–MeOH), the reaction was quenched by the addition of satd aq NaHCO_3_. The mixture was diluted with CHCl_3_ and washed with brine. The organic layer was subsequently dried over Na_2_SO_4_, and concentrated. The residue was purified by silica gel column chromatography (20:1 CHCl_3_–MeOH) to give **40** (8.0 mg, 91%). [α]_D_ −6.3° (c 0.8, CHCl_3_); ^1^H-NMR (600 MHz, CDCl_3_) δ 8.17–7.27 (m, 30 H, 6 Ph), 6.68 (d, 1 H, *J*_2,NH_ = 8.2 Hz, NH^Cer^), 6.21 (d, 1 H, *J*_2,NH_ = 9.0 Hz, NHb), 5.85 (near dt, 1 H, *J*_5,6_ = 6.9 Hz, *J* = 14.4 Hz, *J* = 7.5 Hz, H-5^Cer^), 5.70 (d, 1 H, *J*_3,4_ = 3.4 Hz, H-4d), 5.59 (m, 1 H, H-8g), 5.57 (near t, 1 H, *J*_1,2_ = 8.3 Hz, *J*_2,3_ = 10.3 Hz, H-2d), 5.45 (dd, 1 H, H-3d), 5.41–5.38 (m, 1 H, H-8e), 5.35–5.32 (m, 2 H, H-2a, H-7e), 5.27–5.17 (m, 7 H, H-4a, H-3b, H-2f, H-7g, NHe, H-3^Cer^, H-4^Cer^), 5.09 (d, 1 H, *J*_3,4_ = 2.7 Hz, H-4f), 5.06 (near t, 1 H, *J*_1,2_ = 4.8 Hz, *J*_2,3_ = 5.5 Hz, H-2h), 4.94 (d, 1 H, *J*_5,NH_ =10.3 Hz, NHg), 4.91–4.81 (m, 5 H, H-3c, H-1d, H-4e, H-4g, NHc), 4.74–4.70 (m, 2 H, H-1a, H-3f), 4.62 (d, 1 H, H-1h), 4.45–4.34 (m, 6 H, H-1b, H-1c, H-9e, H-6f, H-9g, H-6h), 4.27–4.23 (m, 3 H, H-2^cer^, H-6a, H-6d), 4.17–3.98 (m, 11 H, H-6'a, H-6c, H-6'c, H-6d, H-5e, H-6e, H-9'e, H-9'g, H-5h, H-6h, H-6'h), 3.88–3.65 (m, 17 H, H-3a, H-2b, H-4b, H-6b, H-6'b, H-2c, H-4c, H-5g, H-3h, H-4h, H-1^Cer^, 2 OCH_3_), 3.60–3.53 (m, 3 H, H-5d, H-6g, H-1^Cer^), 3.50–3.44 (m, 2 H, H-5b, H-5f), 3.29–3.23 (m, 2 H, H-5a, H-5c), 2.56–2.39 (m, 6 H, H-3e*eq*, H-3g*eq*, 2 C(=O)CH_2_), 2.20–1.78 (m, 56 H, 17 Ac, H-3e*ax*, H-6^Cer^, H-6'^Cer^, C(=O)CH_2_^Cer^), 1.62 (t, 1 H, *J*_gem_ = *J*_3ax,4_ = 12.6 Hz, H-3g*ax*), 1.54 (s, 3 H, Ac), 1.51–1.46 (m, 2 H, C(=O)CH_2_CH_2_), 1.42 (s, 3 H, Ac), 1.38–1.20 (m, 50 H, 25 –CH_2_–), 0.88 (m, 6 H, 2 –CH_3_^Cer^); ^13^C-NMR (150 MHz, CDCl_3_) δ 172.9, 171.0, 170.9, 170.8, 170.8, 170.7, 170.6, 170.5, 170.5, 170.3, 170.3, 170.3, 170.1, 170.1, 170.0, 169.9, 169.8, 169.7, 169.7, 168.0, 167.8, 165.7, 165.4, 165.3, 165.2, 165.0, 164.7, 138.5, 134.0, 133.5, 133.3, 133.2, 130.3, 129.8, 129.7, 129.7, 129.6, 129.5, 129.4, 129.0, 129.0, 128.7, 128.6, 128.5, 128.4, 128.4, 128.4, 125.0, 101.7, 101.2, 100.7, 99.8, 99.6, 99.3, 98.5, 96.8. 82.0, 77.5, 75.2, 74.5, 74.2, 73.8, 73.5, 72.9, 72.7, 72.6, 72.5, 72.0, 71.9, 71.7, 71.2, 71.0, 70.9, 70.7, 70.1, 69.9, 69.6, 69.4, 68.6, 67.8, 67.6, 67.4, 67.2, 67.1, 66.6, 66.3, 62.9, 69.4, 68.6, 67.8, 67.6, 67.4, 67.2, 67.1, 66.6, 66.3, 62.9, 62.5, 62.4, 62.0, 61.1, 60.9, 54.2, 53.9, 53.0, 53.0, 50.1, 49.5, 48.8, 37.8, 37.2, 36.4, 32.2, 31.9, 30.4, 29.7, 29.7, 29.7, 29.6, 29.5, 29.5, 29.3, 29.2, 28.8, 25.6, 23.6, 23.2, 23.1, 22.7, 22.5, 21.4, 21.1, 20.8, 20.8, 20.7, 20.6, 20.5, 20.4, 14.1. *m/z* (MALDI): found [M+Na]^+^ 3589.60, C_176_H_231_N_5_O_72_ calcd for [M+Na]^+^ 3589.45. HRMS (ESI): found [1/2M+Na]+ 1806.2182, C176H231N5O72 calcd for [1/2M+Na]+ 1806.2181.



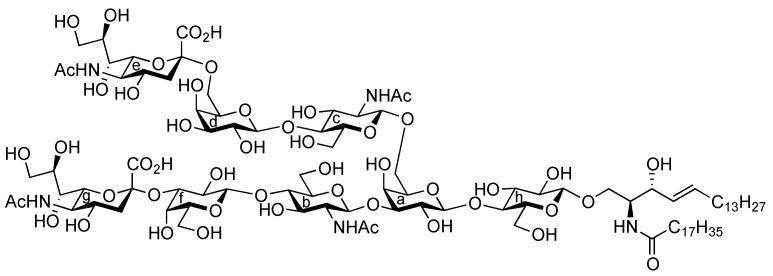



*{3-O-[(5-Acetamido-3,5-dideoxy-D-glycero-α-D-galacto-2-nonulopyranosylonic acid)-β-D-galactopyranosyl-(1→4)-2-acetamido-2-deoxy-β-D-glucopyranosyl]-6-O-[(5-acetamido-3,5-dideoxy-D-glycero-α-D-galacto-2-nonulopyranosylonic acid)-β-D-galactopyranosyl-(1→4)-2-acetamido-2-deoxy-β-D-glucopyranosyl]-β-D-galactopyranosyl}-(1→4)-β-D-glucopyranosyl-(1→1)-(2S,3R,4E)-2-octadecanamido-4-octadecene-1,3-diol* (**1**). To a solution of **40** (8.0 mg, 2.24 μmol) in MeOH/THF (1:1, 1.0 mL) was added NaOMe (28% solution in MeOH, 12.1 μg, 0.672 μmol) at 0 °C. After stirring for 7 d at 40 °C as the reaction was monitored by TLC (6:4:1 CHCl_3_–MeOH–0.2% aq CaCl_2_) and MALDI-TOF MS, water (0.1 mL) was added to the reaction mixture. After stirring for 2 d at 40 °C, the reaction was neutralized with Dowex (H^+^) resin. The resin was filtered through cotton and the filtrate was then evaporated. The residue was purified by gel filtration column chromatography (LH-20) using MeOH as eluent followed by silica gel column chromatography (6:4:1 CHCl_3_–MeOH–H_2_O) to give **1** (3.0 mg, 61%). [α]_D_ −78.8° (c 0.4, MeOH); ^1^H-NMR (600 MHz, 1:1 CD_3_OD–D_2_O) δ 5.73 (m, 1 H, H-5^Cer^), 5.46 (m, 1 H, H-4^Cer^), 2.86 (dd, 1 H, H-3g*eq*), 2.78 (dd, 1 H, H-3e*eq*), 2.20 (t, 2 H, NHCOCH_2_), 2.04 (s, 14 H, H-6^Cer^, H-6'^Cer^, 4 NAc), 1.73 (m, 2 H, H-3g*ax*, H-3e*ax*), 1.58 (m, 2 H, NHCOCH_2_CH_2_), 1.30 (m, 50 H, 25 –CH_2_–), 0.92 (t, 6 H, 2 –CH_3_); ^13^C-NMR (125 MHz, 5:6:0.5 CD_3_OD–D_2_O–CDCl_3_) δ 174.2, 174.1, 173.7, 173.3, 134.1, 128.6, 102.9, 102.3, 102.0, 101.8, 99.9, 79.9, 79.0, 77.8, 76.5, 75.1, 74.6, 74.1, 74.0, 73.5, 73.1, 72.5, 72.2, 72.0, 71.7, 71.6, 71.1, 70.9, 70.6, 70.2, 69.3, 68.7, 68.2, 67.9, 67.8, 67.5, 67.3, 66.8, 62.4, 62.2, 60.4, 59.7, 54.6, 54.5, 52.4, 51.6, 51.5, 48.1, 46.7, 39.7, 39.4, 35.6, 31.6, 31.1, 31.0, 29.0, 28.8, 28.7, 28.6, 28.6, 28.5, 28.4, 25.3, 21.8, 21.6, 21.4, 21.2, 21.1, 12.9. HRMS (ESI): found [M+Na]^−^ 2225.0943, C_98_H_171_N_5_O_49_ calcd for [M+Na]^–^ 2225.0940. 

### 3.2. Materials and Methods for Binding Assay

#### 3.2.1. Virus Preparation

A/Memphis/1/71 (H3N2) and A/Puerto Rico/8/34 (H1N1) were used in this study. The virus was propagated in 11 days old embryonated hen’s eggs. The virus was purified by ultra-centrifugation and stored at −80 °C before use.

#### 3.2.2. Solid-Phase Binding Assay

Virus binding to sialylglycolipids was determined according to a method described previously [28]. Synthetic and authentic gangliosides were 2-fold serially diluted in 100% ethanol from 0.625 to 2.5 pmol/μL. Ten μL of each diluted ganglioside was placed into a well of a polystyrene Universal-BIND^TM^ microplates (flat-bottom, Product# 2503, Corning, Tokyo, Japan) and incubated for approximately 1 h at 37 °C until the ethanol had completely evaporated. gangliosides were covalently immobilized on the surface of plates by exposure for 1 min under ultraviolet irradiation (254 nm) according to the manufacture’s instruction. Each well was blocked for 1 h at room temperature with PBS containing 2% bovine serum albumin. After 3 washes with PBS, the plates were incubated in solutions containing viruses in PBS (128 HA unit/50 μL/well) overnight at 4 °C. After 5 washes with ice-cold PBS, the plates were incubated in a substrate solution containing 40 µM 2'-(4-methylumbelliferyl)-α-D-*N*-acetylneuraminic acid in PBS (50 μL/well) at 37 °C for 60 min to detect virus neuraminidase (NA) activity associated with bound viruses. The reactions were terminated by addition of 500 mM carbonate buffer (pH 10.2) (50 μL/well). Fluorescence intensity of the 4-methylumbelliferone released by viral NAs was measured at 355 nm (excitation) and 460 nm (emission). Triplicate measurements were performed in each assay. The direct virus binding activity was calculated as follows: Virus-binding score = [(average of NA activity of triplicate ganglioside-immobilized wells) − (average of NA activity of triplicate ethanol-treated wells)]/[NA activity of applied virus (128 HA units/50 μL)]. The virus-binding score was expressed as mean score ± SD.

## 4. Conclusions

We efficiently synthesized a novel ganglioside designed as a ligand for influenza A viruses employing the cassette coupling approach between the heptasaccharyl sugar part and the cyclic glucosylceramide moiety. The present study revealed that the cassette approach can be applied to the synthesis of lacto-series gangliosides as well as ganglio-series gangliosides. This success will expand the applicability of the cassette approach to the synthesis of other glycolipids. In addition, we examined the binding activity of the synthesized ganglioside ligand to influenza A viruses. It was found that the synthetic ligand is recognized by Neuα2-3 and 2-6 type viruses, suggesting that a glycan structure containing both the Neuα2-3Gal and Neuα2-6Gal sequences in a single molecule could exist as a natural ligand for influenza A viruses. To identify an actual natural ligand for influenza viruses, the synthesis of a series of gangliosides with both the Neuα2-3Gal and Neuα2-6Gal sequences in a single molecule is currently underway.
